# Supplementary material for: Impact of a pharmacy-led screening and intervention in people at risk of or living with chronic kidney disease in a primary care setting: a cluster randomised trial protocol
Source: BMJ Open. 2023 Dec 20;13(12):e079110. doi: 10.1136/bmjopen-2023-079110 (PMC10748882; doi:10.1136/bmjopen-2023-079110)
Supplement: Supplementary data [file bmjopen-2023-079110supp002.pdf]

Australian Government

Department of Health
Therapeutic Goods Administration

Australian Register of Therapeutic Goods Certificate

Issued to

Australasian Medical & Scientific Ltd

for approval to supply

Instrument/analyser IVDs

**ARTG Identifier**	197482
**ARTG Start Date**	15/05/2012
**Product Category**	Medical Device Included - IVD Class 1
**GMDN**	CT943
**GMDN Term**	Instrument/analyser IVDs
**Intended Purpose**	Instruments used in the quantitative determination of blood parameters in a whole blood specimen including haemoglobin and haematocrit, glucose (including glucose analysis with critically ill patients e.g. ED, ICU, NICU), ketones, creatinine and lactate (including foetal scalp lactate analysis)

Manufacturer Details	Address	Certificate number(s)
Nova Biomedical Corporation	200 Prospect Street<br>WALTHAM , MASSACH USETTS , 02454<br>United States Of America	

ARTG Standard Conditions

The above Medical Device Included - IVD Class 1 has been entered on the Register subject to the following conditions:

· - The inclusion of the kind of device in the ARTG is subject to compliance with all conditions placed or imposed on the ARTG entry. Refer Part 4-5, Division 2 (Conditions) of the Therapeutic Goods Act 1989 and Part 5, Division 5.2 (Conditions) of the Therapeutic Goods (Medical Devices) Regulations 2002 for relevant information.
- Breaching conditions of the inclusion related to the device of the kind may lead to suspension or cancellation of the ARTG entry; may be a criminal offence; and civil penalties may apply.

Products Covered by This Entry

1. Instrument/analyser IVDs

This entry: does not contain System(s)/Procedure Pack(s)

IVD Information

Name	Category Description
StatStrip Lactate Hospital Meter	Point of care testing
Stat Strip Xpress 2 Meter	Point of care testing
StatStrip Xpress-i Glucose and Ketone Meter	Point of care testing
StatStrip Xpress-i Glucose Meter	Point of care testing

Name	Category Description
StatStrip LAC/Hb/Hct Meter	Point of care testing
Nova Statsensor Creatinine Xpress Meter	Point of care testing
StatStrip Glucose Hospital Meter	Point of care testing
StatStrip Hb/Hct Meter	Point of care testing
StatStrip Xpress-i Lactate Meter	Point of care testing
Stat Profile Prime CCS Analyzer	Point of care testing
Nova Statsensor Creatinine Meter	Point of care testing
Stat Profile Prime ABG Analyzer	Point of care testing
StatStrip Glucose and Ketone Hospital Meter	Point of care testing
StatStrip Xpress2 LAC/Hb/Hct Meter	Point of care testing
Stat Profile Prime Plus Analyser	Point of care testing

Product Specific Conditions

No specific conditions have been recorded against this entry.

Therapeutic Goods Administration
PO Box 100, Woden ACT 2606 Australia
Phone: 1800 020 653
Email: info@tga.gov.au

ARTG Identifier: 197482
ARTG Start Date: 15/05/2012

Australian Government
Department of Health and Aged Care
Therapeutic Goods Administration

Australian Register of Therapeutic Goods Certificate

Issued to

Australasian Medical & Scientific Ltd

for approval to supply

Instrument/analyser IVDs

**ARTG Identifier**	427409
**ARTG Start Date**	6/11/2023
**Product Category**	Medical Device Included - IVD Class 1
**GMDN**	CT943
**GMDN Term**	Instrument/analyser IVDs
**Intended Purpose**	Instruments used for in vitro diagnostic use for the quantitative measurement of creatinine and for calculating Estimated Glomerular Filtration Rate (eGFR).

Manufacturer Details	Address	Certificate number(s)
Nova Biomedical Corporation	200 Prospect Street<br>WALTHAM , MASSACH<br>USETTS , 02454<br>United States Of America	

ARTG Standard Conditions

The above Medical Device Included - IVD Class 1 has been entered on the Register subject to the following conditions:

- - The inclusion of the kind of device in the ARTG is subject to compliance with all conditions placed or imposed on the ARTG entry. Refer Part 4-5, Division 2 (Conditions) of the Therapeutic Goods Act 1989 and Part 5, Division 5.2 (Conditions) of the Therapeutic Goods (Medical Devices) Regulations 2002 for relevant information.
  - Breaching conditions of the inclusion related to the device of the kind may lead to suspension or cancellation of the ARTG entry; may be a criminal offence; and civil penalties may apply.

Products Covered by This Entry

1. Instrument/analyser IVDs

This entry: does not contain System(s)/Procedure Pack(s)

IVD Information

Name	Category Description
Nova Max Pro Creatinine and eGFR Meter	Point of care testing

Product Specific Conditions

No specific conditions have been recorded against this entry.

Therapeutic Goods Administration
PO Box 100, Woden ACT 2606 Australia
Phone: 1800 020 653
Email: info@tga.gov.au

ARTG Identifier: 427409
ARTG Start Date: 6/11/2023
