# Supplementary material for: Impact of a pharmacy-led screening and intervention in people at risk of or living with chronic kidney disease in a primary care setting: a cluster randomised trial protocol
Source: BMJ Open. 2023 Dec 20;13(12):e079110. doi: 10.1136/bmjopen-2023-079110 (PMC10748882; doi:10.1136/bmjopen-2023-079110)
Supplement: Supplementary data [file bmjopen-2023-079110supp004.pdf]

Appendix 7. Trial registration

Status: Registered	Prospectively registered	Up to date

The impact of pharmacy-based screening on identification of unknown kidney disease and a quality use of medicines intervention on inappropriate medication use in people at risk for or living with chronic kidney disease

**Trial Id: ACTRN12622000329763**	**Request Id: 383269**	**Update Trial**	**View Trial**

Date Registered: 22/02/2022	Start date: 01/09/2022	Last approved: 22/02/2022

View data sharing statement
