# Supplementary material for: Impact of a pharmacy-led screening and intervention in people at risk of or living with chronic kidney disease in a primary care setting: a cluster randomised trial protocol
Source: BMJ Open. 2023 Dec 20;13(12):e079110. doi: 10.1136/bmjopen-2023-079110 (PMC10748882; doi:10.1136/bmjopen-2023-079110)
Supplement: Author's manuscript [file bmjopen-2023-079110.draft_revisions.pdf]

BMJ Open is committed to open peer review. As part of this commitment we make the peer review history of every article we publish publicly available.

When an article is published we post the peer reviewers' comments and the authors' responses online. We also post the versions of the paper that were used during peer review. These are the versions that the peer review comments apply to.

The versions of the paper that follow are the versions that were submitted during the peer review process. They are not the versions of record or the final published versions. They should not be cited or distributed as the published version of this manuscript.

BMJ Open is an open access journal and the full, final, typeset and author-corrected version of record of the manuscript is available on our site with no access controls, subscription charges or pay-per-view fees (http://bmjopen.bmj.com).

If you have any questions on BMJ Open's open peer review process please email
info.bmjopen@bmj.com

BMJ Open

The impact of a pharmacy-led screening process and intervention to identify kidney disease and improve medicines use in people at risk of or living with chronic kidney disease

Journal:	*BMJ Open*
Manuscript ID	bmjopen-2023-079110
Article Type:	Protocol
Date Submitted by the Author:	22-Aug-2023

Complete List of Authors:	Tesfaye, Wubshet; The University of Sydney School of Pharmacy<br>Krass, Ines; The University of Sydney School of Pharmacy<br>Sud, Kamal; Westmead Institute for Medical Research, Centre for Transplant and Renal Research; Nepean Hospital, Department of Renal Medicine<br>Johnson, David; The University of Queensland Faculty of Medicine, Centre for Health Services Research; Princess Alexandra Hospital, Department of Kidney and Transplant Services<br>Van, C; The University of Sydney School of Pharmacy<br>Versace, Vincent; Deakin University, Faculty of Health, School of Medicine<br>McMorrow, Rita; The University of Melbourne, Department of General Practice<br>Fethney, Judith; The University of Sydney Susan Wakil School of Nursing and Midwifery, School of Nursing<br>Mullan, Judy; University of Wollongong Graduate School of Medicine<br>Tran, Anh; NHMRC Clinical Trials Centre<br>Robson, Breonny; Kidney Health Australia<br>Vagholkar, Sanjyot; Macquarie University, MQ Health General Practice<br>Kairaitis, Lukas; Blacktown Hospital, Renal Medicine; Western Sydney University School of Medicine<br>Gisev, Natasa; National Drug and Alcohol Research Centre, National Drug and Alcohol Research Centre<br>Fathima, Mariam; The University of Sydney School of Pharmacy<br>Tong, Vivien; The University of Sydney School of Pharmacy<br>Coric, Natali; The University of Melbourne Department of General Practice<br>Castelino, Ronald Lynel; The University of Sydney School of Pharmacy; Blacktown Hospital, Pharmacy

Keywords:	Primary Health Care, Clinical Trial, NEPHROLOGY

SCHOLARONE™ Manuscripts

The impact of a pharmacy-led screening process and intervention to identify kidney disease and improve medicines use in people at risk of or living with chronic kidney disease.

Wubshet Tesfaye (wubshet.tesfaye@sydney.edu.au),[1] Ines Krass (ines.krass@sydney.edu.au),[1] Kamal Sud (Kamal.Sud@health.nsw.gov.au),[2,3] David W Johnson (David.Johnson2@health.qld.gov.au),[4,5] Connie Van (connie.van@sydney.edu.au),[1] Vincent Versace (vincent.versace@deakin.edu.au),[6] Rita McMorrow (rita.mcmorrow@unimelb.edu.au),[7] Judith Fethney (judith.fethney@sydney.edu.au),[8] Judy Mullan (jmullan@uow.edu.au),[9] Anh Tran (a.tran@sydney.edu.au),[10] Breonny Robson (Breonny.Robson@kidney.org.au),[11] Sanjyot Vagholkar (sanjyot.vagholkar@mqhealth.org.au),[12] Lukas Kairaitis (Lukas.Kairaitis@health.nsw.gov.au),[13,14] Natasa Gisev (n.gisev@unsw.edu.au),[15] Mariam Fathima (mariam.fathima@sydney.edu.au),[1] Vivien Tong (vivien.tong@sydney.edu.au),[1] Natali Coric (n.coric@unimelb.edu.au),[7] Ronald L Castelino(ronald.castelino@sydney.edu.au)[1,16]

1. Sydney Pharmacy School, The University of Sydney, NSW, Australia.
2. Sydney Medical School, Faculty of Medicine and Health, The University of Sydney, NSW, Australia.
3. Nepean Kidney Research Centre, Department of Renal Medicine, Nepean Hospital, Nepean and Blue Mountains Local Health District, NSW, Australia.
4. Centre for Health Services Research, Faculty of Medicine, University of Queensland, QLD, Australia.
5. Department of Kidney and Transplant Services, Princess Alexandra Hospital, Brisbane, QLD, Australia.
6. Deakin Rural Health, Faculty of Health, Deakin University, VIC, Australia.
7. General Practice and Primary Care, Faculty of Medicine, Dentistry and Health Sciences, The University of Melbourne, VIC, Australia.
8. Faculty of Medicine and Health, Susan Wakil School of Nursing and Midwifery, The University of Sydney, NSW, Australia.
9. Graduate School of Medicine, University of Wollongong, NSW, Australia.
10. NHMRC Clinical Trials centre, The University of Sydney, NSW, Australia.
11. Kidney Health Australia.
12. MQ Health General Practice, Macquarie University, NSW, Australia.
13. Department of Renal Medicine, Blacktown Hospital, NSW, Australia.
14. School of Medicine, Western Sydney University, NSW, Australia.
15. National Drug and Alcohol Research Centre, University of New South Wales Sydney, NSW, Australia.
16. Pharmacy Department, Blacktown Hospital, NSW, Australia.

Corresponding author

Assoc. Professor Ronald Castelino | PhD MPharm, BPharm, BCGP
Senior Lecturer | The University of Sydney School of Pharmacy
A15, Science Rd, Camperdown NSW 2006
e: Ronald.Castelino@sydney.edu.au
p: +61 2 9351 0609 | m: ++61406360715

For peer review only

The impact of a pharmacy-led screening process and intervention to identify kidney disease and improve medicines use in people at risk of or living with chronic kidney disease.

ABSTRACT

Introduction: Chronic kidney disease (CKD) is increasingly recognised as a growing global public health problem. Early detection and management can significantly reduce the loss of kidney function. The proposed trial aims to evaluate the impact of a community pharmacy-led intervention combining CKD screening and medication review on CKD detection and quality use of medicines (QUM) for patients with CKD. We hypothesise that the proposed intervention will enhance detection of newly diagnosed CKD cases and reduce potentially inappropriate medications use by people at risk of or living with CKD.

Methods and analysis: This study is a multi-centre, pragmatic, two-level cluster randomised controlled trial (cRCT) which will be conducted across different regions in Australia. Clusters of community pharmacies from geographical groups of co-located postcodes will be randomised. The project will be conducted in 122 community pharmacies distributed across metropolitan and rural areas. The trial consists of two arms: (i) Control Group: A risk assessment using the QKidney® CKD risk assessment tool, and (ii) Intervention Group: a risk assessment using the QKidney® CKD plus Point-of-Care Testing (POCT) for kidney function markers (serum creatinine and estimated glomerular filtration rate), followed by a QUM service. The primary outcomes of the study are the proportion of patients newly diagnosed with CKD at the end of the study period (12 months); and rates of changes in the number of medications considered problematic in kidney disease (number of medications prescribed at inappropriate doses based on kidney function and/or number of nephrotoxic medications) over the same period. Secondary outcomes include proportion of people on potentially inappropriate medications, types of recommendations provided by the pharmacist (and acceptance rate by general practitioners), proportion of people who were screened, referred, and took up the referral to visit their general practitioners, economic and other patient-centred outcomes.

Ethics and dissemination: The trial protocol has been approved by the Human Research Ethics Committee at the University of Sydney (2022/044) and the findings of the study will be presented at scientific conferences and published in peer-reviewed journal(s).

Trial registration number: Australian New Zealand Clinical Trials Registry (ACTRN12622000329763).

***Keywords**: Chronic kidney disease, screening, point-of-care testing, quality use of medicines, cluster randomised controlled trial, pharmacy-led intervention*

For peer review only - http://bmjopen.bmj.com/site/about/guidelines.xhtml

Strengths and limitations of the study

- This is the first large-scale and multicentre cluster randomised controlled trial to explore the effectiveness of pharmacy-led point-of-care testing to identify people with previously undiagnosed CKD, with the view to ensure quality use of medicines.
- Enhanced detection of CKD through community pharmacy-led primary care intervention may enable earlier use of diagnostic and treatment strategies, reducing the burden of progressive CKD.
- The use of geographical sampling plan that accounts for metropolitan and rural settings will provide a representative data.
- Improvement in medication use will depend on the appropriateness of the recommendations as well as their rate of acceptance and implementation of recommendation by GPs and patients.
- The current community pharmacy reform around 60-day dispensing in Australia may impact recruitment of and participation by pharmacies.

BACKGROUND

Chronic kidney disease (CKD) is a growing public health problem, which affects over 10% of the adult population worldwide.[1] CKD is defined as sustained kidney damage (presence of proteinuria) and/or reduced kidney function (estimated glomerular filtration rate (eGFR) of <60mL/min/1.73m^2) lasting for at least 3 months.[2] The global prevalence of CKD is estimated at 9.1%, with high-income countries such as the United States, Canada, and Australia are the most affected.[1] There has been a substantial increase in the incidence of people with kidney failure, with people requiring kidney replacement therapies such as dialysis and transplantation increased by 43% over the past three decades.[3] CKD is more prevalent in people with socioeconomic disadvantage, ethnic minorities in the UK and the US, as well as First Nations peoples in Australia, New Zealand and Canada.[1,2]

Inappropriate use of medications in people with CKD ranges from 24% to 70%.[4-9] In Australian primary care, approximately 35% of patients are prescribed potentially inappropriate medications based on their kidney function.[10] As many as 32% of adults with CKD receive inappropriate medications upon hospital admission.[11] The inappropriate use of medications in people with CKD can increase risk of adverse drug events, including hospitalisations and death, and many of these events are potentially preventable by optimising their use.[12]

Inadequate recognition of CKD in primary care is one factor that can contribute to the increased risks of adverse drug events. An Australian study reported that although all patients fulfilled the criteria for a standard CKD definition, only 20% of patients had a documented CKD diagnosis.[11] Overall, less than a quarter of patients attending general practices with a pathology report consistent with CKD had a term representing kidney disease in their electronic medical records.[13] Evidence-based guidelines recommend early recognition, referral and management

For peer review only - http://bmjopen.bmj.com/site/about/guidelines.xhtml

as the most effective approaches to slowing the progression of CKD.[14-17] If CKD is detected early and managed appropriately, loss of kidney function can be reduced by as much as 50%.[16]

The first Australian Strategic Kidney Disease Action Plan was developed with the aim of preventing and managing CKD through effective use of research, evidence and data.[2] As part of this plan, collaboration with pharmacists is proposed as a key strategy to improve patient safety.[4] Community pharmacies represent an underutilised resource with significant potential to contribute to the early identification and management of individuals at risk of CKD. There are over 5,600 community pharmacies spread across metropolitan, regional and remote Australia.[18] Each person in Australia visits community pharmacies 18 times a year (compared to 6 times for GP visits),[19,20] highlighting the opportunity to reach diverse communities, including those in remote regions with limited access to other health services.

Despite mounting evidence to support pharmacists' involvement in disease screening and medication management,[21,22] community pharmacists currently have limited role in CKD care. Hence, we propose a novel and yet practical intervention to utilise the community pharmacy setting to screen for CKD using Point-of-Care Testing (POCT) coupled with a quality use of medicines (QUM) intervention. The use of POCT by pharmacists has been previously evaluated and demonstrated in diabetes and in patients taking warfarin.[23-25] Small observational studies from the Netherlands and Canada also explored the effectiveness of using POCT in pharmacies for early detection of CKD,[26,27] while an Australian study evaluated the feasibility of a pharmacist-led CKD screening.[28]

The Australian study involving 24 community pharmacies and 389 patients found that 52% of screened participants had moderate or high risk of developing CKD, but only 53% of them subsequently had kidney function tests via a pathology provider.[28] The pilot work highlighted issues such as poor referral uptake by GPs, lack of service remuneration, and absence of objective measurements for kidney function.[28,29] To address these challenges, the proposed trial comprises an improved model of care that involves a more effective collaboration between pharmacists and GPs, including incentives for both, and implementing a POCT for objective measurements of kidney function.

Trial Objectives

The overarching aim is to evaluate the effectiveness of pharmacy-led screening in enhanced detection of previously unknown CKD cases and quality use of medicines (QUM) intervention in optimising medication use for patients with CKD. Specifically, the aim is to:

For peer review only - http://bmjopen.bmj.com/site/about/guidelines.xhtml

- Determine whether implementation of a community pharmacy-led screening using POCT will improve the detection of previously undiagnosed CKD compared to risk assessment alone.
- Evaluate if the community pharmacy-led QUM service informed by POCT will improve the appropriateness of medication use in people with CKD.

Hypotheses

We hypothesise that the use of a POCT with CKD risk assessment (POCT + QKidney® Risk Assessment: Intervention Group) in community pharmacies will result in (i) a significantly improved detection of previously unknown CKD cases, and (ii) reduced use of potentially inappropriate medications when compared to CKD risk assessment alone (QKidney® Risk Assessment: Control Group). Additionally, the incorporation of POCT in conjunction with the QKidney® Risk Assessment will lead to enhanced utilisation of kidney beneficial medications (i.e., those that slow kidney disease progression) and a long-term economic benefit.

METHODS

We developed this protocol in line with the Standard Protocol Items: Recommendations for Interventional Trials guidelines (SPIRIT statement, **Appendix 1**).[30]

Study setting and design overview.

This study is a multi-centre, pragmatic, two-level cluster randomised controlled trial (cRCT). Community pharmacies (clusters) are the unit of randomisation and screening participants are the unit of analysis. The project will be conducted in community pharmacies across three Australian states of New South Wales (NSW), Victoria (VIC), and Queensland (QLD) and later expanded to the Australian Capital Territory.

Community pharmacies will be selected from geographical groups of co-located postcodes to form clusters within the top 50 kidney disease hotspot areas identified by Kidney Health Australia.[14] Co-located pharmacies within a cluster represent a local community that will all receive the same service model to reduce contamination bias. Pairs of clusters matched by characteristics such as location and socioeconomic status[31] will be randomly allocated to the intervention or control groups. Objective geographical access is described using the Modified Monash Model (MMM). Consistent with the Australian Government Department of Health and Aged Care's definition used in the Rural Health Multidisciplinary Training Program,[32,33] the MMM for this study was broadly stratified into Metropolitan (MM1) and Rural (MM2-7) categories. Given there were no postcodes classified as 'MM6 – Remote Communities' or

For peer review only - http://bmjopen.bmj.com/site/about/guidelines.xhtml

'MM7 – Very Remote Communities' identified within the top 50 kidney disease hotspots, the Rural categorisation was constrained to MM2-5. The number of community pharmacies to be targeted is determined based on the respective population sizes of the states, with proportional pharmacy distributions across Metropolitan and Rural settings. Therefore, based upon the population of the states and the percentage contribution of each state, a total of 122 community pharmacies will be recruited from the three states with 1:1 control:intervention allocation, representing 50 pharmacies from New South Wales (38 Metropolitan vs 12 Rural), 40 from Victoria (30 vs 10) and 32 from Queensland (20 vs 12). Given the proximity of some of the NSW pharmacies, we have expanded our recruitment to include pharmacies in the Australian Capital Territory.

After the identification of the initial round of postcodes, these were stratified, and colour coded by the geospatial expert (VV) based on socioeconomic status and remoteness to ensure a balanced representation. The allocation into the intervention and control arms was randomised centrally at the University of Sydney by the project manager.

Selection of Community Pharmacies: Community pharmacies within identified postcodes will be identified by triangulating sources such as *"Yellow Pages*", "Google" and the pharmacy finder in the Pharmacy Guild's website. All identified pharmacies that are approved to dispense Pharmaceutical Benefits Scheme (PBS) medicines (Australia's universal medicine subsidy program); have a separate or private counselling area; accredited by an approved pharmacy accreditation program; have FRED/Z dispensing software will be eligible to participate in the study. Of note, pharmacies without FRED/Z systems but have capacity for manual data entry may be considered for inclusion. Prior to participating in the trial, eligible pharmacies will be required to complete an online training and a baseline survey on existing practices on any screening and QUM services in different chronic diseases including CKD. All identified pharmacies will be contacted by the research team to participate in the study and, if consented to participate, provided with relevant training and onboarding information.

Sample Size: Sample size selection and estimation was performed based on the CKD diagnosis. Using the GRT sample size calculator,[34] it is estimated that the intervention will increase diagnosis from the current CKD prevalence of 10% to between 15% – 20%, an increase of 5% – 10%. Sample size is estimated based on a 5% increase in diagnosis of CKD. With 80% power, alpha of 0.05 and intraclass correlation coefficient (ICC) = 0.05, at least 57 clusters are required per arm. Based on our sampling model, cluster size has been increased to 61 per arm to account for attrition. With an average number of 30 participants per cluster, the total sample size to be

For peer review only - http://bmjopen.bmj.com/site/about/guidelines.xhtml

targeted is 3,660. Based on previous study[11] that showed a reduction of inappropriate medications in CKD from 32.3% to 17.5% (an approximate reduction of 15%), with 90% power, alpha = 0.05 and ICC of 0.05 and a cluster size of 30 per cluster, 19 clusters in each arm are required (38 clusters in all) with a sample size of 1,140 for the other primary outcome, i.e., change in inappropriate medication use. The sample size required for the diagnosis outcome is, therefore, sufficient to cover the sample size required for the medication changes outcome.

Selection of Pharmacists

Pharmacists who work in the selected community pharmacies will be eligible to participate if:

- They are currently registered by the Australian Health Practitioner Regulation Agency.
- Satisfactorily complete a Continuing Professional Development (CPD)-accredited online training course and assessment.
- Agree to follow procedures outlined in the trial protocol.
- Demonstrate competency in the use of POCT testing using the device supplied for the trial (intervention group).

The content for the CPD online training course, which is accredited by the Pharmaceutical Society of Australia, was developed by the research team for online delivery through Medcast Pty Ltd. The online training consists of three or four self-directed eLearning modules depending on the study arm: (i) Trial Overview; (ii) identification and enrolment of participants; (iii) pharmacy-led CKD screening using QKidney® risk assessment tool and POCT; and (iv) QUM service. Pharmacists in both the intervention (will take all four modules) and control (modules 1-3) groups will be required to complete an assessment at the end of online training, which comprises multiple choice questions and requires an 80% pass mark to obtain a certificate of completion. Pharmacists can claim up to 5 CPD points, with the possibility for claiming additional CPD points for the time spent on conducting services related to the trial. The online learning modules will be evaluated to explore pharmacists' experience and overall level of satisfaction, the relevance of the training to their practice and whether the stated learning objectives were achieved.

Pharmacists in the intervention group will receive additional training on the POCT device used for this study (Statsensor, Nova Biomedical, Waltham MA, USA).[35] This analyser uses a finger prick sample of whole blood (1.2 μL) to measure creatinine within 30 seconds. In-built software then allows calculation of the eGFR or estimated creatinine clearance (CrCL) via the CKD

For peer review only

Epidemiology Collaboration (CKD-EPI) or Cockcroft-Gault equations. The device has been approved by the Therapeutic Goods Administration (TGA) (**Appendix 2**) and is used in radiology and cardiac catheterisation services to minimise risk of contrast-induced nephropathy as well as in oncology and emergency departments to expedite patient care. All participating pharmacies will receive the online training modules specific to the intervention group including on POCT and are expected to demonstrate competence in obtaining the blood sample using the device.

Selection of participants eligible for screening

Participants who are eligible for screening must:

- Be between the age of 35 to 74 years.
- Be at an increased risk of developing CKD due to one or more of the following parameters: ≥60 years of age, hypertension, diabetes, established cardiovascular disease, obesity (BMI ≥ 30 kg/m^2), current or former smoker, family history of kidney disease, prior acute kidney injury, Aboriginal & Torres Strait Islander origin.
- Not have kidney failure or not be receiving kidney replacement therapy.
- Not have a terminal illness.
- Not be pregnant.
- Be able to make independent decisions about their health.

Eligibility will be determined based on self-report by the prospective screening participant and based on the pharmacist's judgement of the participant being able to make independent decisions about their health.

Clinical protocol and recruitment procedure

The clinical protocol for the trial is summarised in **Figure 1**, which illustrates each of the steps involved in recruitment, screening, QUM service and referral to GP. Promotional materials including flyers, posters and eligibility card for self-assessment will be provided to each participating pharmacy. Each pharmacy will assign one person to serve as the study champion, while all pharmacy staff members, including pharmacists, pharmacy interns and pharmacy assistants and technicians will be encouraged to support the recruitment. The expected duration of the participant recruitment is 6 months per pharmacy. The POCT kit in the intervention group pharmacies will be rotated across pharmacies over 12 months to ensure each pharmacy has at least 6 months to recruit 30 participants.

For peer review only - http://bmjopen.bmj.com/site/about/guidelines.xhtml

Screening participants will be recruited through the targeted community pharmacies. The recruitment process involves the pharmacists discussing the study and confirming participant eligibility. The pharmacist will manage potential participants using a purpose-built online software application hosted on a cloud-based server at The University of Sydney. This will ensure the integration of the recruitment process, including the delivery of intervention into the pharmacy recording platform and thereby automate key processes, such as risk assessment, medication history taking, referral letter production and follow-up. Also, direct storage of data in the cloud server will ensure no data are lost due to any systemic issues arising from individual pharmacies.

Following identification of screening participants, those who are eligible and willing to participate will be provided with the participant information statement to read and be asked to sign two consent forms. The first one is to participate in the study and to access their medical records from the GP and My Health Record (a secure online platform used in Australia used to store key healthcare information – this will facilitate pharmacists' verification of clinical information when they make referral to GPs). The second consent will be used to release their Medicare data from the Australian Government Department of Health.

Following recruitment, screening participants in the ***control*** group, if they are known CKD patients (stages 1-4; based on self-report), will be provided with lifestyle advice and counselling on medications to avoid in kidney disease along with a Kidney Health Australia factsheet on 'Looking after yourself with kidney disease'. All other control group patients will have their risk of developing CKD computed using the QKidney® risk calculator,[36] or based on blood pressure levels, if they have at least one of the known CKD risk factors. Participants will be advised of an appropriate action based on their calculated risk. All participants will be provided with lifestyle advice using a range of educational fact sheets, sourced from Kidney Health Australia resources, targeting their risk factors for CKD.

Following recruitment, participants in the ***intervention*** group will be divided into two groups.

1. Those with pre-existing CKD (Stages 1-4; based on self-report) and a SCr and eGFR measured within the last 3 months (accessed via My Health Record) will have their dispensing history and medication event summaries (My Health Record) reviewed to determine the presence of any medications considered problematic in kidney disease. Participants with inappropriate medication, doses or medication combinations based on their kidney function will have recommendations to adjust the doses of medications or

For peer review only - http://bmjopen.bmj.com/site/about/guidelines.xhtml

cease contraindicated medications (QUM service) to their GP. Additionally, participants who, because of their kidney function, are eligible but not receiving potentially beneficial medications (e.g., renin angiotensin system inhibitors or sodium glucose co-transporter 2 inhibitors) will have recommendations provided to their GPs to consider starting these medications. The pharmacists will be encouraged to upload their recommendations on the My Health Record platform as event summaries. Participants with no SCr/eGFR recorded in the last 3 months will have their kidney function determined using POCT followed by the QUM service.

2. Participants with no prior history of CKD and at least one CKD risk factor will have their risk of developing CKD computed using the QKidney® risk calculator. Participants who return with a moderate to high risk (>3% of developing moderate to severe CKD over five years) will have their kidney function determined using a POCT device for SCr and eGFR and will have their dispensing history reviewed to determine the presence of any medications considered problematic in kidney disease. Participants with any inappropriate dosages or contraindicated medications as per their kidney function will have recommendations to their GP to adjust the doses of medications/ceasing contraindicated medications or medication interactions (QUM service). Also, the recommendations may include introduction of potentially beneficial medications as highlighted above. The pharmacists will be encouraged to upload their recommendations on My Health Record as event summaries.

Screened participants in the intervention group are expected to attend a pharmacy session lasting between 20 and 45 mins with the trained pharmacist to assess and collect basic demographic and clinical/medical information (e.g., height, weight, blood pressure, medications dispensed), and to apply the QKidney® Risk assessment and POCT protocol and operating procedures. The time it takes for the overall procedure depends on the individual participant's circumstances, such as the presence of pre-existing CKD or level of risk of developing it, type and number of medications involved as well as the need for screening referral to a GP. Similarly, participants in the control group will be expected to attend a pharmacy session at baseline lasting between 20 and 30 mins for the relevant risk assessment using the QKidney® Risk tool and to provide relevant education and resources pertinent to their level of risk and risk factors for CKD.

Participants in the intervention group will be followed up by pharmacists/research project officers via phone or SMS at six weeks to determine the status of the recommended follow-up

For peer review only - http://bmjopen.bmj.com/site/about/guidelines.xhtml

with the GP and to re-advise GP review if participants have not yet made an appointment with their GP. All participants will be followed-up by pharmacists/research officers at 6 months and 12 months to determine the number of medications changed and new cases of CKD diagnosed (in participants with no prior history).

Data collection, monitoring, management, and reporting:

Data collection is purpose designed and will be facilitated by a highly secured and password protected online software application. Study protocols, templates for assessing CKD risk, links to the QKidney® risk calculator, template for GP referral, QKidney® health advice, template for QUM service will be included in the software along with specific guides to support access and familiarisation. Intervention group pharmacies will also be able to record the POCT results in the software. This will maximise trial efficiency and reduce the burden on pharmacists.

Adherence to the study protocol will be regularly monitored for accuracy and completeness by the project manager and research officers using the online study website and site visits. In addition, project officers will use a range of communication strategies to ensure that the community pharmacists are kept informed of the latest developments of the study, and to provide ongoing support to the pharmacists and these include regular phone calls and bulk emailing options to allow an early action where pharmacies require additional support with the study.

Data collection including pre-screening, screening, and post-screening activities and the respective timepoints are outlined in **Table 1**.

Table 1. Study procedures and timepoints

Procedure	Baseline	6 weeks	3 months	6 months	12 months
**Pre-screening/screening activities**					
Site randomisation	X				
Information Sheet	X				
Pharmacist/Participant consent	X				
Initial screening	X				
Pharmacy survey (current services)	X				
Inclusion/Exclusion Criteria - Review	X				
Demography & Medical History	X				
**Outcome assessment**					
Proportion of people with CKD				X	X
Changes in # of PIMs				X	X
% of participants with CKD risk factors	X			X	X
QKidney® Risk profiles	X				
% Participants referred to GP	X				

For peer review only - http://bmjopen.bmj.com/site/about/guidelines.xhtml

% Participants taking up referral with GP				X	X
Phone/SMS Reminder (PO/RA)		X			
Other outcomes (Health-related quality of life, well-being)	X				X
**Study evaluations**					
Economic evaluation (health resource use and costs)					X
Pharmacist Midtrial Interview			X		X
Pharmacist End of Trial interview					X
GP satisfaction					X
Participant knowledge/satisfaction survey					X
Study compliance audit	X		X	X	X

Outcomes

Primary Outcome Measures

- Proportion of patients newly diagnosed with CKD at the end of the study period.
- Changes in the number of medications considered problematic in kidney disease (number of medications prescribed at inappropriately high dose, number of contraindicated medications or detrimental drug interactions).

Secondary Outcome Measures

- Proportion of screening participants with inappropriate medications (number of medications at inappropriate high dose based on the kidney function and number of contraindicated medications)
- Proportion of eligible participants without potentially beneficial medications (e.g., renin angiotensin system inhibitors or sodium glucose co-transporter 2 inhibitors)
- Type and number of proposed medication adjustments recommended by pharmacists and acceptance rates by physicians.
- Proportion of participants with CKD risk factors.
- Proportion of participants screened (Process indicator).
- Participants' QKidney® risk profiles.
- Percentage of participants referred to their GP.
- Percentage of participants who take up referral with their GP.
- Number of hospitalisations due to medication-related events.
- Cost effectiveness.
- Health-related Quality of life, consumer well-being and satisfaction.

For peer review only

For peer review only - http://bmjopen.bmj.com/site/about/guidelines.xhtml

Both the intervention and control group participants will be followed-up by the project officers/pharmacists at 6 and 12 months to determine if they have had any additional blood tests, and changes to their medications, and hospitalisations.

Primary outcomes will be determined based on data collected from GPs. Health-related quality of life and overall participant well-being will be measured using the EuroQoL-5D-5L, which consists of a descriptive system and a visual analogue scale.[37] The EQ-5D-5L is scored on a 0-1 utility scale, where 0=death and 1=full and complete health. Unit values will be informed by Australian population estimates.[38] A minimally important difference of 0.07 (7%) change in utility on the EQ-5D-5L scale is considered to be clinically meaningful.[39]

Screening participants' satisfaction with the service and willingness to pay for the service will be determined by a telephone questionnaire, which will include items relating to service approval (5-point Likert scale); and open-ended questions to elicit reasons for approval and satisfaction ratings. Screening participants' willingness to pay for the service will also be determined including the maximum amount. Satisfaction of pharmacists (at midtrial and end of trial) with the screening and QUM services will be assessed using electronic surveys. The GPs involved in the care of the participants will also be sent a survey at the end of the trial to determine their satisfaction and any effects on their practice.

A purposive sample of screening participants and GPs will be invited for a follow-up interview based on their responses to the surveys. The aim will be to identify issues that may limit the effectiveness of the proposed intervention. Pharmacist satisfaction with the service will be determined by inviting pharmacists who delivered the intervention to attend focus group discussions. The topics covered will include overall pharmacist experience of the screening program, including consumer perspectives, GP communications, business impact and implications for future implementation.

For peer review only

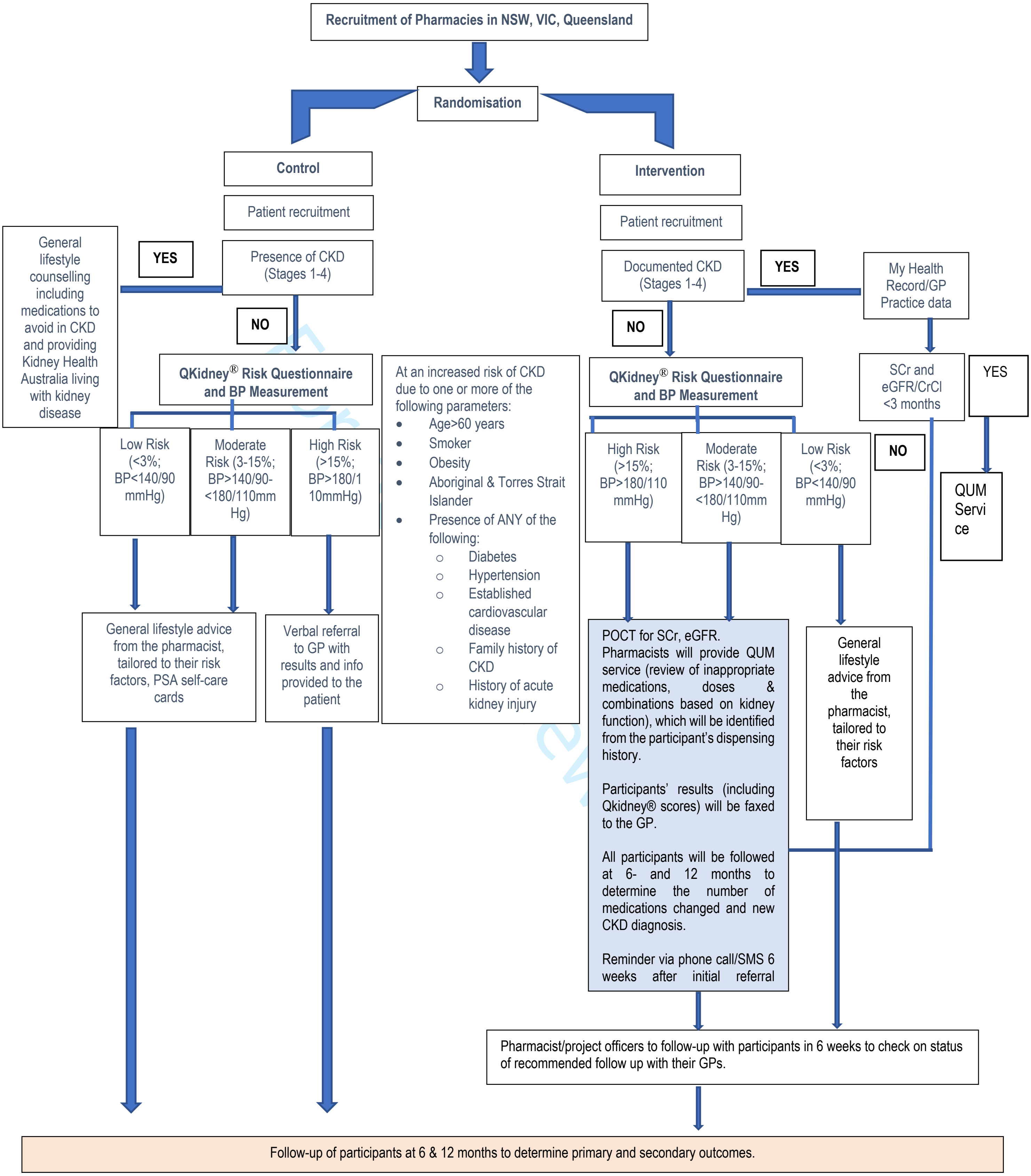

Figure 1. Procedures of recruitment of participants

For peer review only - http://bmjopen.bmj.com/site/about/guidelines.xhtml

Data analysis plan

All data will be reported following the Consolidated Standards of Reporting Trial (CONSORT) guidelines.[40] Baseline clinical and demographic characteristics will be summarised using frequencies and percentages for categorical variables, and means, standard deviations, medians, interquartile ranges, and minimum and maximum values for continuous variables, as appropriate. All statistical analyses for the primary and secondary outcomes will follow the intention to treat (ITT) principle. The ITT population will include all randomised participants based on their initial assignment to either intervention or control group and include any participants who prematurely withdraw or poorly comply with the protocol.

All primary and secondary analyses will use Generalized Estimating Equations (GEE) with pharmacy as the unit of clustering, using the model appropriate for the distribution of the outcome. The analysis of the primary outcome (reported as a binary variable 0 = not diagnosed with CKD, 1 = diagnosed with CKD) will use a logistic regression within a model to test the effectiveness of the intervention. Analysis will firstly be unadjusted (including as fixed factors group membership and the stratification variable, region), followed by analysis adjusted for relevant baseline covariates (age, gender, blood pressure measures, body mass index, socioeconomic status, and location of pharmacies), as well as the fixed factors from the unadjusted analysis. Binary secondary outcomes such as percentage of participants with CKD risk factors, those having a GP review, and those with potentially inappropriate medications will be analysed using a similar approach as that for the primary outcome.

Additional outcomes including numbers of recommendations made by pharmacists, numbers of medication-related hospitalisations or acceptance rates by GPs will be analysed using negative binomial regression (or Poisson regression, depending on distribution of the outcome) or logistic regression and will include unadjusted and adjusted analyses as per the primary outcome.

For humanistic outcomes such as participant's health-related quality of life, as well as outcomes on GP satisfaction and participant knowledge/satisfaction, we will use linear regression. All analyses will be presented using unadjusted and adjusted models as per the primary outcome. For proportion of participants screened in the trial (process indicator), we will use the Mantel-Haenszel extension of a Chi Square Test of Independence.

The proportion of missing data will be determined and assessed for any patterns related to the missingness. Unless otherwise stated, multiple imputation (MI) will be used to impute missing

For peer review only - http://bmjopen.bmj.com/site/about/guidelines.xhtml

values. Missing dichotomous data and categorical data with >2 categories will be assessed using the appropriate MI regression techniques, and missing continuous data will be imputed using chained equations. Sensitivity analyses to explore the effect of imputation will be conducted (e.g., complete case; assuming worst case for the missing outcome observations). All tests will be two sided, with statistical significance set at 0.05 and we will use IBM SPSS Statistics for all analyses unless otherwise specified. There will be no adjustment of the alpha level to account for multiple comparisons because the primary and secondary outcomes are specified, unless otherwise stated to be post hoc.

Study evaluation

A logic model for evaluation of this study is presented in **Figure 2**. The model summarises the activities involved in the development and implementation phases, as well as the projected outcomes. The model will be used to frame our approach to evaluating (1) whether the trial was developed and implemented as intended (fidelity and reach), (2) whether study outcomes were achieved or not, (3) the influence of situational factors on results obtained. Baseline surveys, results from the screening and QUM services, and pharmacists' interviews will be used to assess these activities.

Baseline evaluation

At the beginning of the study, participating pharmacies will be required to complete a baseline survey that includes questions on pharmacy characteristics, existing professional services (remunerated and non-remunerated), and strategies and capacity for implementing these services. The information collected will be used to inform the comparability of the two arms in the trial and health economic analysis. All data entered by participant pharmacists into the study software will be extracted to generate quantitative data to enable the trial process evaluation.

Impact evaluation

The impact of the screening and QUM services will be determined by testing the clinical hypothesis as detailed in the clinical protocol and outcomes sections.

Midtrial interviews

Three months after the implementation of the service in the pharmacy, a mix of pharmacists from high and low performance groups in both arms will be invited to participate in an interview to provide feedback to identify challenges, opportunities, barriers, and facilitators to service implementation. This will enable us to provide support to pharmacies and adapt where appropriate.

For peer review only - http://bmjopen.bmj.com/site/about/guidelines.xhtml

End-of-trial interviews

At the end of the study, pharmacists from participating pharmacies representing a mixture of both high recruitment and low recruitment, will be interviewed to gain feedback on the service implementation in the pharmacy, identifying barriers and facilitators to implementing the trial in the pharmacy, perceptions of sustainability and level of engagement by the local GPs in the referral process.

All study participants will receive a survey to determine their quality of life and wellbeing. Referred participants will also receive a survey to determine their level of satisfaction and willingness to pay for the provided service. A random sample of 20% non-referred participants will be surveyed to determine their overall experience and level of satisfaction with the service. A random sample of 10% of GPs who had patients referred to them during the trial will be invited to respond to a telephone/electronic questionnaire to determine their overall experience and level of satisfaction with the service.

All surveys will be created on Research Electronic Data Capture (REDCap), a secure web-based application designed to support data capture for research studies hosted at The University of Sydney.[41] Qualitative interviews will comprise open-ended questions that prompt discussion on a wide range of topics including overall experience with the study, consumer feedback, interaction with GPs and impact of the trial in their pharmacy.

For peer review only - http://bmjopen.bmj.com/site/about/guidelines.xhtml

INPUTS	ACTIVITIES	OUTPUTS	SHORT-TERM OUTCOMES	ANTICIPATED LONG-TERM OUTCOMES
**User and Stakeholders**<br>Consumers (patients)<br>Community pharmacists<br>Community pharmacy staff<br>GPs and practice staff<br><br>**Technology Components**<br>Electronic templates for screening and QUM services, including referral templates, Qkidney® risk and results.<br><br>**Training**<br>Online CPD training program developed for participating pharmacists.<br><br>**Other resources**<br>Material resources (e.g., promotional material (flyers, posters for display etc), Stat sensor POCT for SCr and eGFR/CrCL.	**Preliminary**<br>Project steering committee and Expert panel convened to provide advice on trial implementation; and how to support engagement with GPs, Primary Health Networks, recruit, retain participants, and community pharmacies.<br><br>Recruit pharmacies.<br><br>Implementation of the cRCT.<br><br>**Pharmacists' intervention**<br>Initial training.<br>Implementation planning.<br>Recruitment of eligible participants.<br>Referral of participants with undiagnosed CKD and/or inappropriate medications as per kidney function to the GP according to the clinical protocol.<br><br>Follow up of patients at 6 and 12 months.<br>Regular report of trial progress.<br><br>**Maintenance**<br>Maintain relationship with pharmacies, pharmacists, and other stakeholders.	Improved rates of detection of people with undiagnosed CKD<br><br>Improved use of medicines in patients with kidney disease<br><br>Improved awareness of lifestyle measures to reduce CKD risk.<br><br>Improved collaboration between pharmacists and GPs	Will a pharmacy-led screening and QUM service improve identification of undiagnosed CKD and improve the use of medications?<br><br>**Primary Outcomes**<br>Proportion of newly diagnosed CKD patients<br><br>Proportion of medication changes<br><br>**Secondary Outcomes**<br>Proportion of people with inappropriate medications<br><br>Proportion of people with chronic kidney disease<br><br>Proportion of people screened.<br><br>QKidney® risk profiles<br><br>% Of people referred to their GP<br><br>% Who take up referral with their GP.<br><br>Hospitalisations due to medication-related events.<br><br>Quality of life and other humanistic outcomes such as well-being and consumer satisfaction.<br><br>Cost effectiveness.	Reduced incidence of kidney failure.<br><br>Reduced medication-related events, hospital admissions and mortality related to CKD.<br><br>Guide health policy to efficient use of primary health care resources.

Figure 2. Pharmacy based screening and QUM study logic model.

Economic Evaluation

Health service utilisation will be measured through Medicare and Pharmaceutical Benefits Schemes (MBS and PBS) data linkage, to quantify the symptom-specific and total health service resource use and costs at 12 months, by allocated group. The Consolidated Health Economic Evaluation Reporting Standards (CHEERS) guidelines (**Appendix 3**) will be followed when reporting the health economic evaluation.[42]

Costs

Cost-effectiveness of the intervention will be calculated from a health service provider perspective considering costs incurred by the pharmacists and the government (PBS/MBS) but will not include costs borne by screening participants (e.g., through co-payments charged by GPs). Healthcare resource use including GP visits, specialist visits, diagnostic tests, medicine, hospitalization, emergency admissions will be collected from MBS/PBS history, medication prescription and dispense records, and previous hospital discharge summaries from My Health Record. Unit costs will be collected based on MBS/PBS item numbers, Australian Refined Diagnosis Related Groups (AR-DRG) and Triage codes. Measured costs will include pharmacist time to facilitate data collection at each pharmacy.

Costs analysis

For peer review only

The cost per new CKD case diagnosis for each group will be estimated and compared. We will assess whether the average cost of the new CKD case diagnosis in the intervention group is lower or higher than the average cost of the new CKD case diagnosis in control group. We hypothesise that there is likely to be a difference in average costs per new CKD case diagnosis across the sites in the same cluster and across the clusters from the metropolitan and rural settings. This is due to the differences in sample size, local unit prices of equipment, staff costs and different established local practices.

Differences in overall mean costs between the two groups will be analysed using Generalised Linear Mixed Model. The distribution of residuals from the regression model will be examined and a decision will be made as to whether the costs should be logarithm transformed. Any extreme costs will be carefully examined to decide if they should be considered as extreme outliers and should be trimmed (removed) from the analysis. It is anticipated there will be some missing data. Missing costs data will be examined to ascertain whether it is missing completely at random (MCAR), missing at random (MAR), or missing not at random (MNAR). Simple and multiple imputation methods will be considered based on the pattern of missingness.[43]

Effectiveness

Based on the EQ-5D-5L, utility weight will be calculated via Australian algorithm, which will be used to obtain quality adjusted life-years (QALY).

Within-trial cost-effectiveness analysis

Incremental cost effectiveness ratios (ICERs) that allow analysis of incremental costs of intervention group versus control group per one QALY gained will be calculated.

Modelled cost-effectiveness analysis

The modelled economic evaluation would investigate the benefits of early diagnosis of CKD cases and the associated prevention/delay of the CKD complications like use of kidney replacement therapies or mortality in the long term. The modelled economic evaluation will include the long-term costs of treatment, regular laboratory investigations, treatment of adverse events, hospital admissions and other relevant costs.

The long-term improved outcomes are hypothesised to translate into a cost reduction and offset the increased costs associated with adding pharmacy-led screening to no screening, for CKD case diagnosis. The modelled economic evaluation will also use published results of long-term observational studies to derive assumptions for the decision analytic modelling and to populate

For peer review only - http://bmjopen.bmj.com/site/about/guidelines.xhtml

the model. A 5% discount rate will be applied to both costs and benefits.[44] The uncertainty about results of economic evaluation will be explored using a non-parametric bootstrapping method.

Patient and public involvement

The study will engage key stakeholders in designing, conducting, and reporting the research, as shown in Figure 2. Input from consumers and Kidney Health Australia will be actively sought for sharing research outcomes.

Ethics and dissemination

The study will be conducted in compliance with the International Conference on Harmonisation Good Clinical Practice guidelines, the World Medical Declaration of Helsinki and all amendments, and the Protocol and Protocol Amendments.

The protocol, participant information sheets, consent forms and other relevant case report forms have all been approved by The University of Sydney Human Research Ethics Committee (2022/044). The trial protocol is also registered with the Australian New Zealand Clinical Trials Registry (ACTRN12622000329763).[45] The trial registration is provided in **Appendix 4**. Study findings will be disseminated in plain language and peer-reviewed forums, including at conferences, via publication in medical journals, and to participants, study staff, clinicians, and patient groups, via direct approaches and a variety of traditional and electronic media.

DISCUSSION

The proposed study presents a protocol for the first large-scale cluster randomised trial to evaluate the effectiveness of community pharmacy-led CKD screening and medication management in improving early disease detection and QUM. The sampling plan primarily targets kidney disease hotspot areas identified by Kidney Health Australia, but also accounts for access to services (i.e., metropolitan and rural locations). Within Australia, socio-economic status is lower in rural and remote areas relative to metropolitan areas.[32] The inclusion of MM2-MM5 locations is also relevant to First Nations communities – these areas overlap much of Inner and Outer Australia where 44% of First Nations people reside.[32,46] The study is expected to raise awareness among both pharmacists and at-risk individuals about CKD and its risk factors, potentially leading to the early detection of previously unknown CKD cases. Further, by promoting the safe and effective use of medicines, the study findings will potentially lead to reduced adverse drug events and medication-related hospital admissions in people at risk of, or who live with, CKD. Collectively, the study will generate evidence to support the translation

For peer review only - http://bmjopen.bmj.com/site/about/guidelines.xhtml

of new preventive and public health measures into practice and implement an innovative approach to improving the quality and cost-effectiveness of preventive healthcare intervention.

Effective and sustainable primary care interventions aimed at the early detection and management of CKD have the potential to not only improve health outcomes but also significantly reduce costs associated with extensive hospital services required in later stages, mainly due to the need for regular dialysis or a kidney transplant, which is estimated to cost the Australian economy $5.7 billion a year.[47] More importantly, recent modelling has shown that investing in early detection of kidney disease can save the Australian economy about $10.2 billion over the next 20 years.[47] Pharmacists are well placed to play a role in the early detection, education, and referral of individuals at elevated risk of CKD. By offering screening and QUM services, community pharmacists can contribute to health promotion and education. As integral members of primary healthcare, community pharmacists serve as an important channel for delivering these types of services. Further, their involvement in CKD care contributes towards improving the health status of Australians, including those from lower socioeconomic groups who are disproportionately affected by CKD; increasing public knowledge and awareness of CKD and its risk factors; expanding public health roles for community pharmacists; and enhancing the roles of pharmacists as an integral part of the health workforce. This aligns with Australia's Primary Health Care 10 Year Plan, which promotes better utilisation of primary health care workforce, including allied health professionals and pharmacists.[48]

There is widespread agreement that an effective program is needed to support the identification and better management of individuals with early stages of CKD. The subsequent adoption of such a program, if shown to be successful on a larger scale and in routine practice in Australia, has enormous potential to reduce the population levels of risk and hence reduce preventable healthcare expenditures due to the need for expensive treatments in advanced disease. The adoption of the program will also enhance the effectiveness of currently remunerated pharmacists' QUM services such as MedsCheck and Diabetes MedsCheck, which aim to improve patients' understanding of their medications and ultimately patient outcomes.

By targeting a representative sample of community pharmacies matched by location (rurality) and socioeconomic status,[49] the study is anticipated to represent a wide range of demographics and regions in Australia. Some of the limitations anticipated during recruitment have been previously described and, while it may not be possible to avoid them fully, we will implement proper incentives to the participating pharmacies, pharmacists, and GPs. One major limitation

For peer review only - http://bmjopen.bmj.com/site/about/guidelines.xhtml

of implementing such programmes is the lack of kidney function information availability, which impacts pharmacist's ability to identify and intervene in medication-related problems in people with CKD. Using POCT for determining kidney function markers in community pharmacies, the proposed study aims to fill this gap and promote pharmacists' contributions to CKD care.

This trial introduces an innovative approach by leveraging existing healthcare resources like My Health Record for a more streamlined referral and medication management purposes. This will improve the utilisation of available resources and maximises their potential. Further, the trial employs an integration-based software system that works with selected pharmacy dispensing systems. This integration is expected to provide a more efficient decision support system for medication review and referral, contributing to improved overall patient care. In addition to exploring community pharmacies' readiness to incorporate technological solutions to address QUM issues, the study will shed light on potential barriers and enablers to scalability and sustainability of such systems.

In summary, this research presents a unique opportunity for community pharmacists to engage in health promotion and education of people who are at risk of or living with CKD. Given current existing chronic disease services in community pharmacies, our research will incorporate pharmacists in CKD screening and ensure quality medicines use in people who are at risk of or living with CKD. This is in line with the recommended clinical practice guidelines and National Strategic Plan that highlight the importance of early identification and intervention to slow the progression of CKD and occurrence of complications through a multidisciplinary primary care approach.[2,50]

FUNDING. This work was supported by the Medical Research Future Fund of the Australian Government primary healthcare initiative (MRFQI00008) and conducted collaboratively between The Universities of Sydney, Melbourne, Queensland, and Kidney Health Australia. The funding body has no role in the design of the study as well as collection, analysis and interpretation of data and manuscript write-up.

COMPETING INTERESTS. David Johnson has received consultancy fees, research grants, speaker's honoraria and travel sponsorships from Baxter Healthcare and Fresenius Medical Care, consultancy fees from Astra Zeneca, Bayer, and AWAK, speaker's honoraria from ONO and Boehringer Ingelheim & Lilly, and travel sponsorships from Ono and Amgen. He is a current recipient of an Australian National Health and Medical Research Council Leadership

For peer review only

Investigator Grant. Kamal Sud has received consultancy fees and speaker's honoraria from Baxter Healthcare and speaker's honoraria from Roche. He is on the medical advisory board of Fresenius Medical Care for Australia and New Zealand.

For peer review only

REFERENCES

1. Hill NR, Fatoba ST, Oke JL, et al. Global prevalence of chronic kidney disease–a systematic review and meta-analysis. *PloS one*. 2016;11(7):e0158765.
2. Kidney Health Australia 2019. National Strategic Action Plan for Kidney Disease. Accessed 20/03/2023, https://kidney.org.au/get-involved/advocacy/national-strategic-action-plan-for-kidney-disease
3. Cockwell P, Fisher L-A. The global burden of chronic kidney disease. *The Lancet*. 2020;395(10225):662-664.
4. Fink J, Joy M, St Peter W, Wahba I. Finding a common language for patient safety in CKD. *Clin J Am Soc Nephrol*. 2012;7(4):689-695.
5. Hu KT MA, Stevenson FT. Calculation of the estimated creatinine clearance in avoiding drug dosing errors in the older patient. *Am J Med Sci*. 2001;322(3):133-136.
6. Khanal A PG, Castelino RL, Jose MD. Potentially inappropriate prescribing of renally cleared drugs in elderly patients in community and aged care settings *Drugs Aging*. 2015;32(5):391-400.
7. Long CL RM, Price DW, Magid DJ. Compliance with dosing guidelines in patients with chronic kidney disease. *Ann Pharmacother*. 2004;38(5):853-858.
8. Papaioannou A CJ, Campbell G, Bedard M. Assessment of adherence to renal dosing guidelines in long-term care facilities. *J Am Geriatr Soc*. 2000;48(11):1470-1473.
9. Yap C DD, Thompson J, Baker D. Medication dosing errors for patients with renal insufficiency in ambulatory care. *Jt Comm J Qual Patient Saf*. 2005;31(9):514-521.
10. Castelino R, Saunder T, Kitsos A, et al. Quality Use Of Medicines In Patients With Chronic Kidney Disease. *BMC Nephrol*. 2020;21(1):216.
11. Doody HK PG, Watson D, Castelino RL. Retrospective evaluation of potentially inappropriate prescribing in hospitalized patients with renal impairment. *Curr Med Res Opin*. 2015;31(3):525-535.
12. Tesfaye WH, Castelino RL, Wimmer BC, Zaidi STR. Inappropriate prescribing in chronic kidney disease: A systematic review of prevalence, associated clinical outcomes and impact of interventions. *Int J Clin Pract*. Jul 2017;71(7):e12960. doi:10.1111/ijcp.12960
13. Kitsos A, Peterson, G., Jose, M., Khanam, M., Castelino, R., Radford, J. Variation in Documenting Diagnosable Chronic Kidney Disease in General Medical Practice: Implications for Quality Improvement and Research. *J Prim Care Community Health*. 2019;doi:10:2150132719833298
14. Kidney Health Australia. State of the Nation 2016. Kidney Health Week. Chronic Kidney Disease Hot Spots. Accessed 2/2/2021, https://kidney.org.au/uploads/resources/state-of-the-nation-kidney-health-week-2016-chronic-kidney-disease-hot-spots.pdf
15. Fox CH BA, Zayas LE, McClellan W, Murray B. . Primary care physicians' knowledge and practice patterns in the treatment of chronic kidney disease: an Upstate New York Practice-based Research Network (UNYNET) study. *J Am Board Fam Med*. 2006;19(1):54-61.
16. Johnson D. Evidence-based guide to slowing the progression of early renal insufficiency. *Intern Med J*. 2004;34(1-2):50-57.

For peer review only - http://bmjopen.bmj.com/site/about/guidelines.xhtml

17. Nagykaldi Z, Mold J, Aspy C. Practice facilitators: a review of the literature. *Fam Med*. 2005;37(8):581-588.
18. Duckett S, Romanes D. Submission to Review of Pharmacy Remuneration and Regulation. 2016. *Grattan Institute*. Accessed 26/07/2022. https://www1.health.gov.au/internet/main/publishing.nsf/Content/review-pharmacy-remuneration-regulation-submissions-cnt-5/$file/208-2016-09-23-grattan-institute-submission.pdf
19. NAB Pharmacy Survey. Pharmacy Guild of Australia. Accessed 12/05/2023, https://www.guild.org.au/__data/assets/pdf_file/0014/112055/NAB-Pharmacy-Report-2021.pdf
20. Australians are visiting their GP more and more. 12/05/2023, https://www1.racgp.org.au/newsgp/professional/australians-are-visiting-their-gp-more-and-more.
21. Mc Namara K, Alzubaidi H, Jackson JK. Cardiovascular disease as a leading cause of death: how are pharmacists getting involved? *Integr Pharm Res Pract*. 2019/12/31 2019;8:1-11. doi:10.2147/IPRP.S133088
22. San-Juan-Rodriguez A, Newman TV, Hernandez I, et al. Impact of community pharmacist-provided preventive services on clinical, utilization, and economic outcomes: An umbrella review. *Prev Med*. 2018/10/01/ 2018;115:145-155. doi:https://doi.org/10.1016/j.ypmed.2018.08.029
23. Krass I, Carter R, Mitchell B, et al. Pharmacy Diabetes Screening Trial: protocol for a pragmatic clusterrandomised controlled trial to compare three screening methods for undiagnosed type 2 diabetes in Australian community pharmacy. *BMJ Open*. 2017;doi:10.1136/bmjopen-2017-017725
24. Jackson SL PG, House M, Bartlett T. Point-of-care monitoring of anticoagulant therapy by rural community pharmacists: Description of successful outcomes. *Aust J Rural Health*. 2004;12(5):197-200.
25. Krass I, Carter R, Mitchell B, et al. Pharmacy diabetes screening trial (PDST): Outcomes of a national clustered RCT comparing three screening methods for undiagnosed type 2 diabetes (T2DM) in community pharmacy. *Diabetes Res Clin Pract*. 2023/03/01/ 2023;197:110566. doi:https://doi.org/10.1016/j.diabres.2023.110566
26. Geerts AF, De Koning FH, De Vooght KM, Egberts AC, De Smet PA, van Solinge WW. Feasibility of point-of-care creatinine testing in community pharmacy to monitor drug therapy in ambulatory elderly patients. *J Clin Pharm Ther*. Oct 2013;38(5):416-22. doi:10.1111/jcpt.12081
27. Donovan J, Al Hamarneh YN, Bajorek B, Papastergiou J, Tsuyuki RT. Community pharmacist identification of chronic kidney disease using point-of-care technology: A pilot study. *Can Pharm J*. Mar-Apr 2020;153(2):84-87. doi:10.1177/1715163520902495
28. Gheewala PA, Peterson GM, Zaidi STR, Jose MD, Castelino RL. Evaluation of a chronic kidney disease risk assessment service in community pharmacies. *Nephrology*. Mar 2019;24(3):301-307. doi:10.1111/nep.13247
29. Gheewala P, Peterson G, Zaidi S, Jose M, RL C. Australian Community Pharmacists' Experience of Implementing a Chronic Kidney Disease Risk Assessment Service. . *Prev Chronic Dis*. 2018;15:e81. doi:10.5888/pcd15.170485

For peer review only - http://bmjopen.bmj.com/site/about/guidelines.xhtml

30. Chan A-W, Tetzlaff JM, Altman DG, et al. SPIRIT 2013 Statement: Defining Standard Protocol Items for Clinical Trials. *Ann Intern Med*. 2013/02/05 2013;158(3):200-207. doi:10.7326/0003-4819-158-3-201302050-00583
31. Socio-Economic Indexes for Areas. Australian Bureau of Statistics. Accessed 3/02/2021, https://www.abs.gov.au/ausstats/abs@.nsf/mf/2033.0.55.001
32. Versace VL, Skinner TC, Bourke L, Harvey P, Barnett T. National analysis of the Modified Monash Model, population distribution and a socio-economic index to inform rural health workforce planning. https://doi.org/10.1111/ajr.12805. *Aust J Rural Health*. 2021/10/01 2021;29(5):801-810. doi:https://doi.org/10.1111/ajr.12805
33. Beks H, Walsh S, Alston L, et al. Approaches used to describe, measure, and analyze place of practice in dentistry, medical, nursing, and allied health rural graduate workforce research in Australia: a systematic scoping review. *Int J Environ Res Public Health*. 2022;19(3):1438.
34. GRT Sample Size Calculator - Research Methods Resources | National Institutes of Health (NIH). Accessed 3/2/2021, https://researchmethodsresources.nih.gov/rmr-calculator/build/#/step1
35. Nova Biomedical. Novo StatSensor. Point of Care Creatinine and eGFR Analyzer. Nova Biomedical. http://www.novabio.us/statstrip-creatinine/
36. QKidney® - Risk Calculator. ClinRisk Ltd. Accessed 12/05/2023, http://www.qkidney.org/index.php
37. Herdman M GC, Lloyd A, Janssen M, Kind P, Parkin D, Bonsel G, Badia X. Development and preliminary testing of the new five-level version of EQ-5D (EQ-5D-5L). *Qual Life Res*. 2011;20:1727-36.
38. Norman R CP, Viney R. A pilot discrete choice experiment to explore preferences for EQ-5D-5L health states. *Appl Health Econ Health Policy*. 2013;11(287-98)(3)
39. Walters SJ BJ. Comparison of the minimally important difference for two health state utility measures: EQ-5D and SF-6D. *Qual Life Res*. 2005;14(6):1523-32.
40. Moher D, Hopewell S, Schulz KF, et al. CONSORT 2010 explanation and elaboration: updated guidelines for reporting parallel group randomised trials. *J Clin Epidemiol*. Aug 2010;63(8):e1-37. doi:10.1016/j.jclinepi.2010.03.004
41. Harris PA TR, Thielke R, et al. Research electronic data capture (REDCap)–a metadata-driven methodology and workflow process for providing translational research informatics support. *J Biomed Inform*. 2009;42:377-81.
42. Husereau D, Drummond M, Petrou S, et al. Consolidated Health Economic Evaluation Reporting Standards (CHEERS) statement. *Bmj*. Mar 25 2013;346:f1049. doi:10.1136/bmj.f1049
43. Glick HA, Doshi JA, Sonnad SS, Polsky D. Economic evaluation in clinical trials. OUP Oxford; 2014.
44. Department of Health H, Community S. *G*uidelines for the pharmaceutical industry on preparation of submissions to the Pharmaceutical Benefits Advisory Committee, including submissions involving economic analyses, August 1992 / Commonwealth Department of Health, Housing and Community Services. Australian Govt. Pub. Service; 1992.
45. Pharmacy-Based Screening and Quality Use of Medicines in Kidney Disease. ANZCTR. 05/04/2022, Accessed 05/04/2022,

For peer review only - http://bmjopen.bmj.com/site/about/guidelines.xhtml

https://www.anzctr.org.au/Trial/Registration/TrialReview.aspx?id=383269&isClinicalTrial=False
46. Beks H, Binder MJ, Kourbelis C, et al. Geographical analysis of evaluated chronic disease programs for Aboriginal and Torres Strait Islander people in the Australian primary health care setting: a systematic scoping review. *BMC Public Health*. 2019/08/14 2019;19(1):1115. doi:10.1186/s12889-019-7463-0
47. Changing the chronic kidney disease landscape: The economic benefits of early detection and treatment. Kidney Health Australia. Accessed 03/07/2023, https://www2.deloitte.com/content/dam/Deloitte/au/Documents/Economics/deloitte-au-economics-kidney-health-australia-report-80323.pdf
48. Future focused primary health care: Australia's Primary Health Care 10 Year Plan 2022-2032 Department of Health. Accessed 01/08/2023, https://www.health.gov.au/sites/default/files/documents/2022/03/australia-s-primary-health-care-10-year-plan-2022-2032-future-focused-primary-health-care-australia-s-primary-health-care-10-year-plan-2022-2032.pdf
49. Australian Bureau of Statistics. Socio-Economic Indexes for Areas. Accessed 3/02/2021, https://www.abs.gov.au/ausstats/abs@.nsf/mf/2033.0.55.001
50. James MT, Hemmelgarn BR, Tonelli M. Early recognition and prevention of chronic kidney disease. *Lancet*. 2010;375(9722):1296-1309.

For peer review only

STANDARD PROTOCOL ITEMS: RECOMMENDATIONS FOR INTERVENTIONAL TRIALS

Appendix 1: SPIRIT 2013 Checklist: Recommended items to address in a clinical trial protocol and related documents*

Section/item	Item No	Description	Addressed on page number
**Administrative information**			
Title	1	Descriptive title identifying the study design, population, interventions, and, if applicable, trial acronym	1
Trial registration	2a	Trial identifier and registry name. If not yet registered, name of intended registry	1 & 20
Protocol version	3	Date and version identifier	V2.0
Funding	4	Sources and types of financial, material, and other support	22
Roles and responsibilities	5a	Names, affiliations, and roles of protocol contributors	22-23
	5b	Name and contact information for the trial sponsor	22
	5c	Role of study sponsor and funders, if any, in study design; collection, management, analysis, and interpretation of data; writing of the report; and the decision to submit the report for publication, including whether they will have ultimate authority over any of these activities	22
	5d	Composition, roles, and responsibilities of the coordinating centre, steering committee, endpoint adjudication committee, data management team, and other individuals or groups overseeing the trial, if applicable (see Item 21a for data monitoring committee)	10 (governance details included in master protocol & available on request)

For peer review only

For peer review only - http://bmjopen.bmj.com/site/about/guidelines.xhtml

Introduction

Background and rationale	6a	Description of research question and justification for undertaking the trial, including summary of relevant studies (published and unpublished) examining benefits and harms for each intervention	2-3
	6b	Explanation for choice of comparators	3-4
Objectives	7	Specific objectives or hypotheses	4
Trial design	8	Description of trial design including type of trial (eg, parallel group, crossover, factorial, single group), allocation ratio, and framework (eg, superiority, equivalence, noninferiority, exploratory)	4

Methods: Participants, interventions, and outcomes

Study setting	9	Description of study settings (eg, community clinic, academic hospital) and list of countries where data will be collected. Reference to where list of study sites can be obtained	4
Eligibility criteria	10	Inclusion and exclusion criteria for participants. If applicable, eligibility criteria for study centres and individuals who will perform the interventions (eg, surgeons, psychotherapists)	7
Interventions	11a	Interventions for each group with sufficient detail to allow replication, including how and when they will be administered	8-10
	11b	Criteria for discontinuing or modifying allocated interventions for a given trial participant (eg, drug dose change in response to harms, participant request, or improving/worsening disease)	NA
	11c	Strategies to improve adherence to intervention protocols, and any procedures for monitoring adherence (eg, drug tablet return, laboratory tests)	10
	11d	Relevant concomitant care and interventions that are permitted or prohibited during the trial	NA
Outcomes	12	Primary, secondary, and other outcomes, including the specific measurement variable (eg, systolic blood pressure), analysis metric (eg, change from baseline, final value, time to event), method of aggregation (eg, median, proportion), and time point for each outcome. Explanation of the clinical relevance of chosen efficacy and harm outcomes is strongly recommended	11-12
Participant timeline	13	Time schedule of enrolment, interventions (including any run-ins and washouts), assessments, and visits for participants. A schematic diagram is highly recommended (see Figure)	14 (Figure 1)

For peer review only - http://bmjopen.bmj.com/site/about/guidelines.xhtml

Sample size	14	Estimated number of participants needed to achieve study objectives and how it was determined, including clinical and statistical assumptions supporting any sample size calculations	6
Recruitment	15	Strategies for achieving adequate participant enrolment to reach target sample size	3-4
**Methods: Assignment of interventions (for controlled trials)**			
Allocation:			
Sequence generation	16a	Method of generating the allocation sequence (eg, computer-generated random numbers), and list of any factors for stratification. To reduce predictability of a random sequence, details of any planned restriction (eg, blocking) should be provided in a separate document that is unavailable to those who enrol participants or assign interventions	5
Allocation concealment mechanism	16b	Mechanism of implementing the allocation sequence (eg, central telephone; sequentially numbered, opaque, sealed envelopes), describing any steps to conceal the sequence until interventions are assigned	NA
Implementation	16c	Who will generate the allocation sequence, who will enrol participants, and who will assign participants to interventions	NA
Blinding (masking)	17a	Who will be blinded after assignment to interventions (eg, trial participants, care providers, outcome assessors, data analysts), and how	NA
	17b	If blinded, circumstances under which unblinding is permissible, and procedure for revealing a participant's allocated intervention during the trial	NA
**Methods: Data collection, management, and analysis**			
Data collection methods	18a	Plans for assessment and collection of outcome, baseline, and other trial data, including any related processes to promote data quality (eg, duplicate measurements, training of assessors) and a description of study instruments (eg, questionnaires, laboratory tests) along with their reliability and validity, if known. Reference to where data collection forms can be found, if not in the protocol	10-13
	18b	Plans to promote participant retention and complete follow-up, including list of any outcome data to be collected for participants who discontinue or deviate from intervention protocols	16-18

For peer review only - http://bmjopen.bmj.com/site/about/guidelines.xhtml

Data management	19	Plans for data entry, coding, security, and storage, including any related processes to promote data quality (eg, double data entry; range checks for data values). Reference to where details of data management procedures can be found, if not in the protocol	NA (included in the master protocol & available on request)
Statistical methods	20a	Statistical methods for analysing primary and secondary outcomes. Reference to where other details of the statistical analysis plan can be found, if not in the protocol	15-16
	20b	Methods for any additional analyses (eg, subgroup and adjusted analyses)	15-16
	20c	Definition of analysis population relating to protocol non-adherence (eg, as randomised analysis), and any statistical methods to handle missing data (eg, multiple imputation)	15-16
**Methods: Monitoring**			
Data monitoring	21a	Composition of data monitoring committee (DMC); summary of its role and reporting structure; statement of whether it is independent from the sponsor and competing interests; and reference to where further details about its charter can be found, if not in the protocol. Alternatively, an explanation of why a DMC is not needed	NA (included in the master protocol)
	21b	Description of any interim analyses and stopping guidelines, including who will have access to these interim results and make the final decision to terminate the trial	NA (included in the master protocol)
Harms	22	Plans for collecting, assessing, reporting, and managing solicited and spontaneously reported adverse events and other unintended effects of trial interventions or trial conduct	NA (included in the master protocol)
Auditing	23	Frequency and procedures for auditing trial conduct, if any, and whether the process will be independent from investigators and the sponsor	10
**Ethics and dissemination**			
Research ethics approval	24	Plans for seeking research ethics committee/institutional review board (REC/IRB) approval	20
Protocol amendments	25	Plans for communicating important protocol modifications (eg, changes to eligibility criteria, outcomes, analyses) to relevant parties (eg, investigators, REC/IRBs, trial participants, trial registries, journals, regulators)	20

Consent or assent	26a	Who will obtain informed consent or assent from potential trial participants or authorised surrogates, and how (see Item 32)	8
	26b	Additional consent provisions for collection and use of participant data and biological specimens in ancillary studies, if applicable	NA
Confidentiality	27	How personal information about potential and enrolled participants will be collected, shared, and maintained in order to protect confidentiality before, during, and after the trial	NA (included in the ethics, master protocol)
Declaration of interests	28	Financial and other competing interests for principal investigators for the overall trial and each study site	22
Access to data	29	Statement of who will have access to the final trial dataset, and disclosure of contractual agreements that limit such access for investigators	NA (included in the ethics, master protocol)
Ancillary and post-trial care	30	Provisions, if any, for ancillary and post-trial care, and for compensation to those who suffer harm from trial participation	NA
Dissemination policy	31a	Plans for investigators and sponsor to communicate trial results to participants, healthcare professionals, the public, and other relevant groups (eg, via publication, reporting in results databases, or other data sharing arrangements), including any publication restrictions	20
	31b	Authorship eligibility guidelines and any intended use of professional writers	22-23
	31c	Plans, if any, for granting public access to the full protocol, participant-level dataset, and statistical code	22
**Appendices**			
Informed consent materials	32	Model consent form and other related documentation given to participants and authorised surrogates	Available on request.
Biological specimens	33	Plans for collection, laboratory evaluation, and storage of biological specimens for genetic or molecular analysis in the current trial and for future use in ancillary studies, if applicable	NA

For peer review only

*It is strongly recommended that this checklist be read in conjunction with the SPIRIT 2013 Explanation & Elaboration for important clarification on the items. Amendments to the protocol should be tracked and dated. The SPIRIT checklist is copyrighted by the SPIRIT Group under the Creative Commons "Attribution-NonCommercial-NoDerivs 3.0 Unported" license.

For peer review only

For peer review only - http://bmjopen.bmj.com/site/about/guidelines.xhtml

Australian Government

Department of Health
Therapeutic Goods Administration

Australian Register of Therapeutic Goods Certificate

Issued to

Australasian Medical & Scientific Ltd

for approval to supply

Instrument/analyser IVDs

**ARTG Identifier**	197482
**ARTG Start Date**	15/05/2012
**Product Category**	Medical Device Included - IVD Class 1
**GMDN**	CT943
**GMDN Term**	Instrument/analyser IVDs
**Intended Purpose**	Instruments used in the quantitative determination of blood parameters in a whole blood specimen including haemoglobin and haematocrit, glucose (including glucose analysis with critically ill patients e.g. ED, ICU, NICU), ketones, creatinine and lactate (including foetal scalp lactate analysis)

Manufacturer Details	Address	Certificate number(s)
Nova Biomedical Corporation	200 Prospect Street<br>WALTHAM , MASSACH USETTS , 02454<br>United States Of America	

ARTG Standard Conditions

The above Medical Device Included - IVD Class 1 has been entered on the Register subject to the following conditions:

· - The inclusion of the kind of device in the ARTG is subject to compliance with all conditions placed or imposed on the ARTG entry. Refer Part 4-5, Division 2 (Conditions) of the Therapeutic Goods Act 1989 and Part 5, Division 5.2 (Conditions) of the Therapeutic Goods (Medical Devices) Regulations 2002 for relevant information.
- Breaching conditions of the inclusion related to the device of the kind may lead to suspension or cancellation of the ARTG entry; may be a criminal offence; and civil penalties may apply.

Products Covered by This Entry

1. Instrument/analyser IVDs

This entry: does not contain System(s)/Procedure Pack(s)

IVD Information

Name	Category Description
StatStrip Lactate Hospital Meter	Point of care testing
Stat Strip Xpress 2 Meter	Point of care testing
StatStrip Xpress-i Glucose and Ketone Meter	Point of care testing
StatStrip Xpress-i Glucose Meter	Point of care testing

Name	Category Description
StatStrip LAC/Hb/Hct Meter	Point of care testing
Nova Statsensor Creatinine Xpress Meter	Point of care testing
StatStrip Glucose Hospital Meter	Point of care testing
StatStrip Hb/Hct Meter	Point of care testing
StatStrip Xpress-i Lactate Meter	Point of care testing
Stat Profile Prime CCS Analyzer	Point of care testing
Nova Statsensor Creatinine Meter	Point of care testing
Stat Profile Prime ABG Analyzer	Point of care testing
StatStrip Glucose and Ketone Hospital Meter	Point of care testing
StatStrip Xpress2 LAC/Hb/Hct Meter	Point of care testing
Stat Profile Prime Plus Analyser	Point of care testing

Product Specific Conditions

No specific conditions have been recorded against this entry.

Therapeutic Goods Administration
PO Box 100, Woden ACT 2606 Australia
Phone: 1800 020 653
Email: info@tga.gov.au

ARTG Identifier: 197482
ARTG Start Date: 15/05/2012

Appendix 3. CHEERS checklist

CHEERS Checklist
Items to include when reporting economic evaluations of health interventions

The **ISPOR CHEERS Task Force Report**, *Consolidated Health Economic Evaluation Reporting Standards (CHEERS)—Explanation and Elaboration: A Report of the ISPOR Health Economic Evaluations Publication Guidelines Good Reporting Practices Task Force*, provides examples and further discussion of the 24-item CHEERS Checklist and the CHEERS Statement. It may be accessed via the *Value in Health* or via the ISPOR Health Economic Evaluation Publication Guidelines – CHEERS: Good Reporting Practices webpage: http://www.ispor.org/TaskForces/EconomicPubGuidelines.asp

Section/item	Item No	Recommendation	Reported on page No/ line No
**Title and abstract**			
Title	1	Identify the study as an economic evaluation or use more specific terms such as "cost-effectiveness analysis", and describe the interventions compared.	
Abstract	2	Provide a structured summary of objectives, perspective, setting, methods (including study design and inputs), results (including base case and uncertainty analyses), and conclusions.	
**Introduction**			
Background and objectives	3	Provide an explicit statement of the broader context for the study. Present the study question and its relevance for health policy or practice decisions.	
**Methods**			
Target population and subgroups	4	Describe characteristics of the base case population and subgroups analysed, including why they were chosen.	
Setting and location	5	State relevant aspects of the system(s) in which the decision(s) need(s) to be made.	
Study perspective	6	Describe the perspective of the study and relate this to the costs being evaluated.	
Comparators	7	Describe the interventions or strategies being compared and state why they were chosen.	
Time horizon	8	State the time horizon(s) over which costs and consequences are being evaluated and say why appropriate.	
Discount rate	9	Report the choice of discount rate(s) used for costs and outcomes and say why appropriate.	
Choice of health outcomes	10	Describe what outcomes were used as the measure(s) of benefit in the evaluation and their relevance for the type of analysis performed.	
Measurement of effectiveness	11a	*Single study-based estimates:* Describe fully the design features of the single effectiveness study and why the single study was a sufficient source of clinical effectiveness data.	
	11b	*Synthesis-based estimates:* Describe fully the methods used for identification of included studies and synthesis of clinical effectiveness data.	
Measurement and valuation of preference based outcomes	12	If applicable, describe the population and methods used to elicit preferences for outcomes.	
Estimating resources and costs	13a	*Single study-based economic evaluation:* Describe approaches used to estimate resource use associated with the alternative interventions. Describe primary or secondary research methods for valuing each resource item in terms of its unit cost. Describe any adjustments made to approximate to opportunity costs.	
	13b	*Model-based economic evaluation:* Describe approaches and data sources used to estimate resource use associated with model health states. Describe primary or secondary research methods for valuing each resource item in terms of its unit cost. Describe any adjustments made to approximate to opportunity costs.	
Currency, price date, and conversion	14	Report the dates of the estimated resource quantities and unit costs. Describe methods for adjusting estimated unit costs to the year of reported costs if necessary. Describe methods for converting costs into a common currency base and the exchange rate.	
Choice of model	15	Describe and give reasons for the specific type of decision-analytical model used. Providing a figure to show model structure is strongly recommended.	
Assumptions	16	Describe all structural or other assumptions underpinning the decision-analytical model.	
Analytical methods	17	Describe all analytical methods supporting the evaluation. This could include methods for dealing with skewed, missing, or censored data; extrapolation methods; methods for pooling data; approaches to validate or make adjustments (such as half cycle corrections) to a model; and methods for handling population heterogeneity and uncertainty.	
**Results**			
Study parameters	18	Report the values, ranges, references, and, if used, probability distributions for all parameters. Report reasons or sources for distributions used to represent uncertainty where appropriate. Providing a table to show the input values is strongly recommended.	
Incremental costs and outcomes	19	For each intervention, report mean values for the main categories of estimated costs and outcomes of interest, as well as mean differences between the comparator groups. If applicable, report incremental cost-effectiveness ratios.	
Characterising uncertainty	20a	*Single study-based economic evaluation:* Describe the effects of sampling uncertainty for the estimated incremental cost and incremental effectiveness parameters, together with the impact	

Appendix 7. Trial registration

Status: Registered	Prospectively registered	Up to date

The impact of pharmacy-based screening on identification of unknown kidney disease and a quality use of medicines intervention on inappropriate medication use in people at risk for or living with chronic kidney disease

Trial Id: ACTRN12622000329763	Request Id: 383269	Update Trial	View Trial

Date Registered: 22/02/2022	Start date: 01/09/2022	Last approved: 22/02/2022

View data sharing statement

view only

BMJ Open

The impact of a pharmacy-led screening and intervention in people at risk of or living with chronic kidney disease in a primary care setting: A cluster randomised trial protocol

Journal:	*BMJ Open*
Manuscript ID	bmjopen-2023-079110.R1
Article Type:	Protocol
Date Submitted by the Author:	06-Dec-2023

Complete List of Authors:	Tesfaye, Wubshet; The University of Sydney School of Pharmacy<br>Krass, Ines; The University of Sydney School of Pharmacy<br>Sud, Kamal; Westmead Institute for Medical Research, Centre for Transplant and Renal Research; Nepean Hospital, Department of Renal Medicine<br>Johnson, David; The University of Queensland Faculty of Medicine, Centre for Health Services Research; Princess Alexandra Hospital, Department of Kidney and Transplant Services<br>Van, C; The University of Sydney School of Pharmacy<br>Versace, Vincent; Deakin University, Faculty of Health, School of Medicine<br>McMorrow, Rita; The University of Melbourne, Department of General Practice<br>Fethney, Judith; The University of Sydney Susan Wakil School of Nursing and Midwifery, School of Nursing<br>Mullan, Judy; University of Wollongong Graduate School of Medicine<br>Tran, Anh; NHMRC Clinical Trials Centre<br>Robson, Breonny; Kidney Health Australia<br>Vagholkar, Sanjyot; Macquarie University, MQ Health General Practice<br>Kairaitis, Lukas; Blacktown Hospital, Renal Medicine; Western Sydney University School of Medicine<br>Gisev, Natasa; National Drug and Alcohol Research Centre, National Drug and Alcohol Research Centre<br>Fathima, Mariam; The University of Sydney School of Pharmacy<br>Tong, Vivien; The University of Sydney School of Pharmacy<br>Coric, Natali; The University of Melbourne Department of General Practice<br>Castelino, Ronald Lynel; The University of Sydney School of Pharmacy; Blacktown Hospital, Pharmacy

**Primary Subject Heading**:	Health services research
Secondary Subject Heading:	Health economics, Evidence based practice, Pharmacology and therapeutics, Communication, General practice / Family practice
Keywords:	Primary Health Care, Clinical Trial, NEPHROLOGY

For peer review only - http://bmjopen.bmj.com/site/about/guidelines.xhtml

The impact of a pharmacy-led screening and intervention in people at risk of or living with chronic kidney disease in a primary care setting: A cluster randomised trial protocol.

Wubshet Tesfaye (wubshet.tesfaye@sydney.edu.au),[1] Ines Krass (ines.krass@sydney.edu.au),[1] Kamal Sud (Kamal.Sud@health.nsw.gov.au),[2,3] David W Johnson (David.Johnson2@health.qld.gov.au),[4,5] Connie Van (connie.van@sydney.edu.au),[1] Vincent Versace (vincent.versace@deakin.edu.au),[6] Rita McMorrow (rita.mcmorrow@unimelb.edu.au),[7] Judith Fethney (judith.fethney@sydney.edu.au),[8] Judy Mullan (jmullan@uow.edu.au),[9] Anh Tran (a.tran@sydney.edu.au),[10] Breonny Robson (Breonny.Robson@kidney.org.au),[11] Sanjyot Vagholkar (sanjyot.vagholkar@mqhealth.org.au),[12] Lukas Kairaitis (Lukas.Kairaitis@health.nsw.gov.au),[13,14] Natasa Gisev (n.gisev@unsw.edu.au),[15] Mariam Fathima (mariam.fathima@sydney.edu.au),[1] Vivien Tong (vivien.tong@sydney.edu.au),[1] Natali Coric (n.coric@unimelb.edu.au),[7] Ronald L Castelino(ronald.castelino@sydney.edu.au)[1,16]

1. Sydney Pharmacy School, The University of Sydney, NSW, Australia.
2. Sydney Medical School, Faculty of Medicine and Health, The University of Sydney, NSW, Australia.
3. Nepean Kidney Research Centre, Department of Renal Medicine, Nepean Hospital, Nepean and Blue Mountains Local Health District, NSW, Australia.
4. Centre for Health Services Research, Faculty of Medicine, University of Queensland, QLD, Australia.
5. Department of Kidney and Transplant Services, Princess Alexandra Hospital, Brisbane, QLD, Australia.
6. Deakin Rural Health, Faculty of Health, Deakin University, VIC, Australia.
7. General Practice and Primary Care, Faculty of Medicine, Dentistry and Health Sciences, The University of Melbourne, VIC, Australia.
8. Faculty of Medicine and Health, Susan Wakil School of Nursing and Midwifery, The University of Sydney, NSW, Australia.
9. Graduate School of Medicine, University of Wollongong, NSW, Australia.
10. NHMRC Clinical Trials centre, The University of Sydney, NSW, Australia.
11. Kidney Health Australia.
12. MQ Health General Practice, Macquarie University, NSW, Australia.
13. Department of Renal Medicine, Blacktown Hospital, NSW, Australia.
14. School of Medicine, Western Sydney University, NSW, Australia.
15. National Drug and Alcohol Research Centre, University of New South Wales Sydney, NSW, Australia.
16. Pharmacy Department, Blacktown Hospital, NSW, Australia.

Corresponding author

Assoc. Professor Ronald Castelino | PhD MPharm, BPharm, BCGP
Senior Lecturer | The University of Sydney School of Pharmacy
A15, Science Rd, Camperdown NSW 2006
e: Ronald.Castelino@sydney.edu.au
p: +61 2 9351 0609 | m: ++61406360715

For peer review only - http://bmjopen.bmj.com/site/about/guidelines.xhtml

The impact of a pharmacy-led screening and intervention in people at risk of or living with chronic kidney disease in a primary care setting: A cluster randomised trial protocol.

ABSTRACT

Introduction: Chronic kidney disease (CKD) is increasingly recognised as a growing global public health problem. Early detection and management can significantly reduce the loss of kidney function. The proposed trial aims to evaluate the impact of a community pharmacy-led intervention combining CKD screening and medication review on CKD detection and quality use of medicines (QUM) for patients with CKD. We hypothesise that the proposed intervention will enhance detection of newly diagnosed CKD cases and reduce potentially inappropriate medications use by people at risk of or living with CKD.

Methods and analysis: This study is a multi-centre, pragmatic, two-level cluster randomised controlled trial (cRCT) which will be conducted across different regions in Australia. Clusters of community pharmacies from geographical groups of co-located postcodes will be randomised. The project will be conducted in 122 community pharmacies distributed across metropolitan and rural areas. The trial consists of two arms: (i) Control Group: A risk assessment using the QKidney® CKD risk assessment tool, and (ii) Intervention Group: a risk assessment using the QKidney® CKD plus Point-of-Care Testing (POCT) for kidney function markers (serum creatinine and estimated glomerular filtration rate), followed by a QUM service. The primary outcomes of the study are the proportion of patients newly diagnosed with CKD at the end of the study period (12 months); and rates of changes in the number of medications considered problematic in kidney disease (number of medications prescribed at inappropriate doses based on kidney function and/or number of nephrotoxic medications) over the same period. Secondary outcomes include proportion of people on potentially inappropriate medications, types of recommendations provided by the pharmacist (and acceptance rate by general practitioners), proportion of people who were screened, referred, and took up the referral to visit their general practitioners, economic and other patient-centred outcomes.

Ethics and dissemination: The trial protocol has been approved by the Human Research Ethics Committee at the University of Sydney (2022/044) and the findings of the study will be presented at scientific conferences and published in peer-reviewed journal(s).

Trial registration number: Australian New Zealand Clinical Trials Registry (ACTRN12622000329763).

***Keywords**: Chronic kidney disease, screening, point-of-care testing, quality use of medicines, cluster randomised controlled trial, pharmacy-led intervention*

For peer review only - http://bmjopen.bmj.com/site/about/guidelines.xhtml

Strengths and limitations of the study

- This is the first cluster randomised trial to investigate the effect of pharmacy-led screening of CKD.
- The sampling strategy utilised will ensure recruitment of both metropolitan and rural pharmacies to provide representative data.
- Enhanced CKD detection through community pharmacies may also enable earlier commencement of treatment to delay its progression.
- Pharmacist screening may not necessarily generate the anticipated level of referrals and referral uptakes by GPs.
- The current community pharmacy reform around 60-day dispensing in Australia may impact recruitment of, and participation by, pharmacies.

BACKGROUND

Chronic kidney disease (CKD) is a growing public health problem, which affects over 10% of the adult population worldwide [1]. CKD is defined as sustained kidney damage (presence of proteinuria) and/or reduced kidney function (estimated glomerular filtration rate (eGFR) of <60mL/min/1.73m^2) lasting for at least 3 months [2]. The global prevalence of CKD is estimated at 9.1%, with high-income countries such as the United States, Canada, and Australia the most affected [1]. There has been a substantial increase in the incidence of people with kidney failure, with people requiring kidney replacement therapies such as dialysis and transplantation increased by 43% over the past three decades [3]. CKD is more prevalent in people with socioeconomic disadvantage, ethnic minorities in the UK and the US, as well as First Nations peoples in Australia, New Zealand and Canada [1, 2].

Inappropriate use of medications in people with CKD ranges from 24% to 70% [4-9]. In Australian primary care, approximately 35% of patients are prescribed potentially inappropriate medications based on their kidney function [10]. As many as 32% of adults with CKD receive inappropriate medications upon hospital admission [11]. The inappropriate use of medications in people with CKD can increase risk of adverse drug events, including hospitalisations and death, and many of these events are potentially preventable by optimising their use [12].

Inadequate recognition of CKD in primary care is one factor that can contribute to the increased risks of adverse drug events. An Australian study reported that although all patients fulfilled the criteria for a standard CKD definition, only 20% of patients had a documented CKD diagnosis [11]. Overall, less than a quarter of patients attending general practices with a pathology report consistent with CKD had a term representing kidney disease in their electronic

For peer review only - http://bmjopen.bmj.com/site/about/guidelines.xhtml

medical records [13]. Evidence-based guidelines recommend early recognition, referral, and management as the most effective approaches to slowing the progression of CKD [14-17]. If CKD is detected early and managed appropriately, loss of kidney function can be reduced by as much as 50% [16].

The first Australian Strategic Kidney Disease Action Plan was developed with the aim of preventing and managing CKD through effective use of research, evidence and data [2]. As part of this plan, collaboration with pharmacists is proposed as a key strategy to improve patient safety [4]. Community pharmacies represent an underutilised resource with significant potential to contribute to the early identification and management of individuals at risk of CKD. There are over 5,600 community pharmacies spread across metropolitan, regional and remote Australia [18]. Each person in Australia visits community pharmacies 18 times a year (compared to 6 times for GP visits) [19, 20], highlighting the opportunity to reach diverse communities, including those in remote regions with limited access to other health services.

Despite mounting evidence to support pharmacists' involvement in disease screening and medication management [21, 22], community pharmacists currently have limited role in CKD care. Hence, we propose a novel and yet practical intervention to utilise the community pharmacy setting to screen for CKD using Point-of-Care Testing (POCT) coupled with a quality use of medicines (QUM) intervention. The use of POCT by pharmacists has been previously evaluated and demonstrated in diabetes and in patients taking warfarin [23-25]. Small observational studies from the Netherlands, Spain and Canada also explored the effectiveness of using POCT in pharmacies for early detection of CKD and identification of nephrotoxic medication use [26-28], while an Australian study evaluated the feasibility of a pharmacist-led CKD screening [29].

Our pilot work in an Australian context involving 24 community pharmacies and 389 patients found that 52% of screened participants had moderate or high risk of developing CKD, but only 53% of them subsequently had kidney function tests via a pathology provider [29]. The pilot work highlighted issues such as poor referral uptake by GPs, lack of service remuneration, and absence of objective measurements for kidney function [29, 30]. To address these challenges, the proposed trial comprises an improved model of care that involves a more effective collaboration between pharmacists and GPs, including incentives for both, and implementing a POCT for objective measurements of kidney function.

Trial Objectives

For peer review only - http://bmjopen.bmj.com/site/about/guidelines.xhtml

The overarching aim is to evaluate the effectiveness of pharmacy-led screening in enhanced detection of previously unknown CKD cases and quality use of medicines (QUM) intervention in optimising medication use for patients with CKD. Specifically, the aim is to:

- Determine the effectiveness of a community pharmacy-led screening using POCT on the detection of previously undiagnosed CKD compared to risk assessment alone.
- Evaluate the impact of community pharmacy-led QUM service informed by POCT in improving the appropriateness of medication use in people with CKD.
- Investigate the effectiveness of community pharmacy-led CKD screening and QUM service on long-term economic benefits and humanistic outcomes such as health-related quality of life, and consumer well-being and satisfaction.

Hypotheses

We hypothesise that the use of a POCT with CKD risk assessment (POCT + QKidney® Risk Assessment: Intervention Group) in community pharmacies will result in (i) a significantly improved detection of previously unknown CKD cases, and (ii) reduced use of potentially inappropriate medications when compared to CKD risk assessment alone (QKidney® Risk Assessment: Control Group).

Other hypotheses related to the primary hypothesis are that the Intervention Group will be associated with a statistically significant increase in the proportions of those who are referred to the GP, take up referral with the GP, are newly diagnosed with CKD compared with the Control Group.

Additionally, the incorporation of POCT in conjunction with the QKidney® Risk Assessment will lead to enhanced utilisation of kidney beneficial medications (i.e., those that slow kidney disease progression), a long-term economic benefit and improved humanistic outcomes.

METHODS

Study setting and design overview.

This study protocol is a multi-centre, pragmatic, two-level cluster randomised controlled trial (cRCT). The reporting of this protocol has been guided by the checklist outlined in the Standard Protocol Items: Recommendations for Interventional Trials (SPIRIT statement, **Appendix 1**).[31]

For peer review only - http://bmjopen.bmj.com/site/about/guidelines.xhtml

Community pharmacies (clusters) are the unit of randomisation and screening participants are the unit of analysis. The project will be conducted in community pharmacies across three Australian states of New South Wales (NSW), Victoria (VIC), and Queensland (QLD) and later expanded to the Australian Capital Territory.

Community pharmacies will be selected from geographical groups of co-located postcodes to form clusters within the top 50 kidney disease hotspot areas identified by Kidney Health Australia [14]. Co-located pharmacies within a cluster represent a local community that will all receive the same service model to reduce contamination bias. Pairs of clusters matched by characteristics such as location and socioeconomic status [32] will be randomly allocated to the intervention or control groups. Objective geographical access is described using the Modified Monash Model (MMM). Consistent with the Australian Government Department of Health and Aged Care's definition used in the Rural Health Multidisciplinary Training Program [33, 34], the MMM for this study was broadly stratified into Metropolitan (MM1) and Rural (MM2-7) categories. Given there were no postcodes classified as 'MM6 – Remote Communities' or 'MM7 – Very Remote Communities' identified within the top 50 kidney disease hotspots, the Rural categorisation was constrained to MM2-5. The number of community pharmacies to be targeted is determined based on the respective population sizes of the states, with proportional pharmacy distributions across Metropolitan and Rural settings. Therefore, based upon the population of the states and the percentage contribution of each state, a total of 122 community pharmacies will be recruited from the three states with 1:1 control:intervention allocation, representing 50 pharmacies from New South Wales (38 Metropolitan vs 12 Rural), 40 from Victoria (30 vs 10) and 32 from Queensland (20 vs 12). Given the proximity of some of the NSW pharmacies, we have expanded our recruitment to include pharmacies in the Australian Capital Territory.

After the identification of the initial round of postcodes, these were stratified, and colour coded by the geospatial expert (VV) based on socioeconomic status and remoteness to ensure a balanced representation. The allocation into the intervention and control arms was randomised centrally at the University of Sydney by the project manager.

Selection of Community Pharmacies: Community pharmacies within identified postcodes will be identified by triangulating sources such as *"Yellow Pages*", "Google" and the pharmacy finder in the Pharmacy Guild's website. All identified pharmacies that are approved to dispense Pharmaceutical Benefits Scheme (PBS) medicines (Australia's universal medicine subsidy

For peer review only - http://bmjopen.bmj.com/site/about/guidelines.xhtml

program); have a separate or private counselling area; accredited by an approved pharmacy accreditation program; have FRED/Z dispensing software will be eligible to participate in the study. Of note, pharmacies without FRED/Z systems but have capacity for manual data entry may be considered for inclusion. Prior to participating in the trial, eligible pharmacies will be required to complete an online training and a baseline survey on existing practices on any screening and QUM services in different chronic diseases including CKD. All identified pharmacies will be contacted by the research team to participate in the study and, if consented to participate, provided with relevant training and onboarding information.

Sample Size: Sample size selection and estimation was performed based on the CKD diagnosis. Using the GRT sample size calculator [35], it is estimated that the intervention will increase diagnosis from the current CKD prevalence of 10% to between 15% – 20%, an increase of 5% – 10%. Sample size is estimated based on a 5% increase in diagnosis of CKD. With 80% power, alpha of 0.05 and intraclass correlation coefficient (ICC) = 0.05, at least 57 clusters are required per arm. Based on our sampling model, cluster size has been increased to 61 per arm to account for attrition. With an average number of 30 participants per cluster, the total sample size to be targeted is 3,660. Based on previous study [11] that showed a reduction of inappropriate medications in CKD from 32.3% to 17.5% (an approximate reduction of 15%), with 90% power, alpha = 0.05 and ICC of 0.05 and a cluster size of 30 per cluster, 19 clusters in each arm are required (38 clusters in all) with a sample size of 1,140 for the other primary outcome, i.e., change in inappropriate medication use. The sample size required for the diagnosis outcome is, therefore, sufficient to cover the sample size required for the medication changes outcome.

Selection of Pharmacists

Pharmacists who work in the selected community pharmacies will be eligible to participate if:

- They are currently registered by the Australian Health Practitioner Regulation Agency.
- Satisfactorily complete a Continuing Professional Development (CPD)-accredited online training course and assessment.
- Agree to follow procedures outlined in the trial protocol.
- Demonstrate competency in the use of POCT testing using the device supplied for the trial (intervention group).

The content for the CPD online training course, which is accredited by the Pharmaceutical Society of Australia, was developed by the research team for online delivery through Medcast

For peer review only - http://bmjopen.bmj.com/site/about/guidelines.xhtml

Pty Ltd. The online training consists of three or four self-directed eLearning modules depending on the study arm: (i) Trial Overview; (ii) identification and enrolment of participants; (iii) pharmacy-led CKD screening using QKidney® risk assessment tool and POCT; and (iv) QUM service. Pharmacists in both the intervention (will take all four modules) and control (modules 1-3) groups will be required to complete an assessment at the end of online training, which comprises multiple choice questions and requires an 80% pass mark to obtain a certificate of completion. Pharmacists can claim up to 5 CPD points, with the possibility for claiming additional CPD points for the time spent on conducting services related to the trial. The online learning modules will be evaluated to explore pharmacists' experience and overall level of satisfaction, the relevance of the training to their practice and whether the stated learning objectives were achieved.

Pharmacists in the intervention group will receive additional training on the POCT (performed using Nova Statsensor Creatinine Meter or Nova Max Pro Creatinine and eGFR Meter, Nova Biomedical, Waltham MA, USA) [36], devices approved for use by the Therapeutic Goods Administration (TGA) (**Appendix 2**). The analyser in these devices uses a finger prick sample of whole blood (1.2 µL) to measure creatinine within 30 seconds. In-built software then allows calculation of the eGFR or estimated creatinine clearance (CrCL) via the CKD Epidemiology Collaboration (CKD-EPI) or Cockcroft-Gault equations. Such POCT devices are used to determine kidney function used in radiology and cardiac catheterisation services to minimise risk of contrast-induced nephropathy as well as in oncology and emergency departments to expedite patient care. They have also been used in emergency and community pharmacy settings for detection of nephrotoxic medications [28, 37]. All participating pharmacies will receive the online training modules specific to the intervention group including on POCT and are expected to demonstrate competence in obtaining the blood sample using the device.

<u>Selection of participants eligible for screening</u>

Participants who are eligible for screening must:

- Be between the age of 35 to 74 years.
- Be at an increased risk of developing CKD due to one or more of the following parameters: ≥60 years of age, hypertension, diabetes, established cardiovascular disease, obesity (BMI ≥ 30 kg/m^2), current or former smoker, family history of kidney disease, prior acute kidney injury, Aboriginal & Torres Strait Islander origin.
- Not have kidney failure or not be receiving kidney replacement therapy.

For peer review only - http://bmjopen.bmj.com/site/about/guidelines.xhtml

- Not have a terminal illness.
- Not be pregnant.
- Be able to make independent decisions about their health.

Eligibility will be determined based on self-report by the prospective screening participant and based on the pharmacist's judgement of the participant being able to make independent decisions about their health.

Clinical protocol and recruitment procedure

The clinical protocol for the trial is summarised in **Figure 1**, which illustrates each of the steps involved in recruitment, screening, QUM service and referral to GP. Promotional materials including flyers, posters and eligibility card for self-assessment will be provided to each participating pharmacy. Each pharmacy will assign one person to serve as the study champion, while all pharmacy staff members, including pharmacists, pharmacy interns and pharmacy assistants and technicians will be encouraged to support the recruitment. The expected duration of the participant recruitment is 6 months per pharmacy. The POCT kit in the intervention group pharmacies will be rotated across pharmacies over 12 months to ensure each pharmacy has at least 6 months to recruit 30 participants.

Screening participants will be recruited through the targeted community pharmacies. The recruitment process involves the pharmacists discussing the study and confirming participant eligibility. The pharmacist will manage potential participants using a purpose-built online software application hosted on a cloud-based server at The University of Sydney. This will ensure the integration of the recruitment process, including the delivery of intervention into the pharmacy recording platform and thereby automate key processes, such as risk assessment, medication history taking, referral letter production and follow-up. Also, direct storage of data in the cloud server will ensure no data are lost due to any systemic issues arising from individual pharmacies.

Following identification of screening participants, those who are eligible and willing to participate will be provided with the participant information statement to read and be asked to sign two consent forms. The first one is to participate in the study and to access their medical records from the GP and My Health Record (a secure online platform used in Australia used to store key healthcare information – this will facilitate pharmacists' verification of clinical information when they make referral to GPs). The second consent will be used to release their Medicare data from the Australian Government Department of Health.

For peer review only - http://bmjopen.bmj.com/site/about/guidelines.xhtml

Following recruitment, screening participants in the ***control*** group, if they are known CKD patients (stages 1-4; based on self-report), will be provided with lifestyle advice and counselling on medications to avoid in kidney disease along with a Kidney Health Australia factsheet on 'Looking after yourself with kidney disease'. All other control group patients will have their risk of developing CKD computed using the QKidney® risk calculator [38], or based on blood pressure levels, if they have at least one of the known CKD risk factors. Participants will be advised of an appropriate action based on their calculated risk. All participants will be provided with lifestyle advice using a range of educational fact sheets, sourced from Kidney Health Australia resources, targeting their risk factors for CKD.

Following recruitment, participants in the ***intervention*** group will be divided into two groups.

1. Those with pre-existing CKD (Stages 1-4; based on self-report) and a SCr and eGFR measured within the last 3 months (accessed via My Health Record) will have their dispensing history and medication event summaries (My Health Record) reviewed to determine the presence of any medications considered problematic in kidney disease. Participants with inappropriate medication, doses or medication combinations based on their kidney function will have recommendations to adjust the doses of medications or cease contraindicated medications (QUM service) to their GP. Additionally, participants who, because of their kidney function, are eligible but not receiving potentially beneficial medications (e.g., renin angiotensin system inhibitors or sodium glucose co-transporter 2 inhibitors) will have recommendations provided to their GPs to consider starting these medications. The pharmacists will be encouraged to upload their recommendations on the My Health Record platform as event summaries. Participants with no SCr/eGFR recorded in the last 3 months will have their kidney function determined using POCT followed by the QUM service.
2. Participants with no prior history of CKD and at least one CKD risk factor will have their risk of developing CKD computed using the QKidney® risk calculator. Participants who return with a moderate to high risk (>3% of developing moderate to severe CKD over five years) will have their kidney function determined using a POCT device for SCr and eGFR and will have their dispensing history reviewed to determine the presence of any medications considered problematic in kidney disease. Participants with any inappropriate dosages or contraindicated medications as per their kidney function will have recommendations to their GP to adjust the doses of medications/ceasing contraindicated medications or medication interactions (QUM service). Also, the recommendations may

For peer review only - http://bmjopen.bmj.com/site/about/guidelines.xhtml

include introduction of potentially beneficial medications as highlighted above. The pharmacists will be encouraged to upload their recommendations on My Health Record as event summaries.

Screened participants in the intervention group are expected to attend a pharmacy session lasting between 20 and 45 mins with the trained pharmacist to assess and collect basic demographic and clinical/medical information (e.g., height, weight, blood pressure, medications dispensed), and to apply the QKidney® Risk assessment and POCT protocol and operating procedures. The time it takes for the overall procedure depends on the individual participant's circumstances, such as the presence of pre-existing CKD or level of risk of developing it, type and number of medications involved as well as the need for screening referral to a GP. Similarly, participants in the control group will be expected to attend a pharmacy session at baseline lasting between 20 and 30 mins for the relevant risk assessment using the QKidney® Risk tool and to provide relevant education and resources pertinent to their level of risk and risk factors for CKD.

Participants in the intervention group will be followed up by pharmacists/research project officers via phone or SMS at six weeks to determine the status of the recommended follow-up with the GP and to re-advise GP review if participants have not yet made an appointment with their GP. All participants will be followed-up by pharmacists/research officers at 6 months and 12 months to determine the number of medications changed and new cases of CKD diagnosed (in participants with no prior history).

Data collection, monitoring, management, and reporting:

Data collection is purpose designed and will be facilitated by a highly secured and password protected online software application. Study protocols, templates for assessing CKD risk, links to the QKidney® risk calculator, template for GP referral, QKidney® health advice, template for QUM service will be included in the software along with specific guides to support access and familiarisation. Intervention group pharmacies will also be able to record the POCT results in the software. This will maximise trial efficiency and reduce the burden on pharmacists.

Adherence to the study protocol will be regularly monitored for accuracy and completeness by the project manager and research officers using the online study website and site visits. In addition, project officers will use a range of communication strategies to ensure that the community pharmacists are kept informed of the latest developments of the study, and to provide ongoing support to the pharmacists and these include regular phone calls and bulk

For peer review only - http://bmjopen.bmj.com/site/about/guidelines.xhtml

For peer review only

emailing options to allow an early action where pharmacies require additional support with the study.

Data collection including pre-screening, screening, and post-screening activities and the respective timepoints are outlined in **Table 1**. The study is set to take place over two years (March 2023 – May 2025), and will include enrolling pharmacists and patients, 6- and 12-month follow-ups, and report writing.

Table 1. Study procedures and timepoints

Procedure	Baseline	6 weeks	3 months	6 months	12 months
**Pre-screening/screening activities**					
Site randomisation	X				
Information Sheet	X				
Pharmacist/Participant consent	X				
Initial screening	X				
Pharmacy survey (current services)	X				
Inclusion/Exclusion Criteria - Review	X				
Demography & Medical History	X				
**Outcome assessment**					
Proportion of people with CKD				X	X
Changes in # of PIMs				X	X
% of participants with CKD risk factors	X			X	X
QKidney® Risk profiles	X				
% Participants referred to GP	X				
% Participants taking up referral with GP				X	X
Phone/SMS Reminder (PO/RA)		X			
Other outcomes (Health-related quality of life, well-being)	X				X
**Study evaluations**					
Economic evaluation (health resource use and costs)					X
Pharmacist Midtrial Interview			X		X
Pharmacist End of Trial interview					X
GP satisfaction					X
Participant knowledge/satisfaction survey					X
Study compliance audit	X		X	X	X

Outcomes

Primary Outcome Measures

- Proportion of patients newly diagnosed with CKD at the end of the study period.
- Changes in the number of medications considered problematic in kidney disease (number of medications prescribed at inappropriately high dose, number of contraindicated medications or detrimental drug interactions).

Secondary Outcome Measures

For peer review only - http://bmjopen.bmj.com/site/about/guidelines.xhtml

- Proportion of screening participants with inappropriate medications (number of medications at inappropriate high dose based on the kidney function and number of contraindicated medications).
- Proportion of eligible participants without potentially beneficial medications (e.g., renin angiotensin system inhibitors or sodium glucose co-transporter 2 inhibitors).
- The number and characteristics of proposed medication adjustments recommended by pharmacists and acceptance rates by GPs.
- Proportion of participants with CKD risk factors.
- Proportion of participants screened (Process indicator).
- Participants' QKidney® risk profiles.
- Percentage of participants referred to their GP.
- Percentage of participants who take up referral with their GP.
- Number of hospitalisations due to medication-related events.
- Cost effectiveness.
- Health-related Quality of life, consumer well-being and satisfaction.

Both the intervention and control group participants will be followed-up by the project officers/pharmacists at 6 and 12 months to determine if they have had any additional blood tests, and changes to their medications, and hospitalisations.

Primary outcomes will be determined based on data collected from GPs. Health-related quality of life and overall participant well-being will be measured using the EuroQoL-5D-5L, which consists of a descriptive system and a visual analogue scale [39]. The EQ-5D-5L is scored on a 0-1 utility scale, where 0=death and 1=full and complete health. Unit values will be informed by Australian population estimates [40]. A minimally important difference of 0.07 (7%) change in utility on the EQ-5D-5L scale is considered to be clinically meaningful [41].

Screening participants' satisfaction with the service and willingness to pay for the service will be determined by a telephone questionnaire, which will include items relating to service approval (5-point Likert scale); and open-ended questions to elicit reasons for approval and satisfaction ratings. Screening participants' willingness to pay for the service will also be determined including the maximum amount. Satisfaction of pharmacists (at midtrial and end of trial) with the screening and QUM services will be assessed using electronic surveys. The GPs involved in the care of the participants will also be sent a survey at the end of the trial to determine their satisfaction and any effects on their practice.

For peer review only - http://bmjopen.bmj.com/site/about/guidelines.xhtml

For peer review only

A purposive sample of screening participants and GPs will be invited for a follow-up interview based on their responses to the surveys. The aim will be to identify issues that may limit the effectiveness of the proposed intervention. Pharmacist satisfaction with the service will be determined by inviting pharmacists who delivered the intervention to attend focus group discussions. The topics covered will include overall pharmacist experience of the screening program, including consumer perspectives, GP communications, business impact and implications for future implementation.

Data analysis plan

All data will be reported following the Consolidated Standards of Reporting Trial (CONSORT) guidelines [42]. Baseline clinical and demographic characteristics will be summarised using frequencies and percentages for categorical variables, and means, standard deviations, medians, interquartile ranges, and minimum and maximum values for continuous variables, as appropriate. All statistical analyses for the primary and secondary outcomes will follow the intention to treat (ITT) principle. The ITT population will include all randomised participants based on their initial assignment to either intervention or control group and include any participants who prematurely withdraw or poorly comply with the protocol.

All primary and secondary analyses will use Generalized Estimating Equations (GEE) with pharmacy as the unit of clustering, using the model appropriate for the distribution of the outcome. The analysis of the primary outcome (reported as a binary variable 0 = not diagnosed with CKD, 1 = diagnosed with CKD) will use a logistic regression within a model to test the effectiveness of the intervention. Analysis will firstly be unadjusted (including as fixed factors group membership and the stratification variable, region), followed by analysis adjusted for relevant baseline covariates (age, gender, blood pressure measures, body mass index, socioeconomic status, and location of pharmacies), as well as the fixed factors from the unadjusted analysis. Binary secondary outcomes such as percentage of participants with CKD risk factors, those having a GP review, and those with potentially inappropriate medications will be analysed using a similar approach as that for the primary outcome.

Additional outcomes including numbers of recommendations made by pharmacists, numbers of medication-related hospitalisations or acceptance rates by GPs will be analysed using negative binomial regression (or Poisson regression, depending on distribution of the outcome) or logistic regression and will include unadjusted and adjusted analyses as per the primary outcome.

For peer review only

For humanistic outcomes such as participant's health-related quality of life, as well as outcomes on GP satisfaction and participant knowledge/satisfaction, we will use linear regression. All analyses will be presented using unadjusted and adjusted models as per the primary outcome. For proportion of participants screened in the trial (process indicator), we will use the Mantel-Haenszel extension of a Chi Square Test of Independence.

The proportion of missing data will be determined and assessed for any patterns related to the missingness. Unless otherwise stated, multiple imputation (MI) will be used to impute missing values. Missing dichotomous data and categorical data with >2 categories will be assessed using the appropriate MI regression techniques, and missing continuous data will be imputed using chained equations. Sensitivity analyses to explore the effect of imputation will be conducted (e.g., complete case; assuming worst case for the missing outcome observations). All tests will be two sided, with statistical significance set at 0.05 and we will use IBM SPSS Statistics for all analyses unless otherwise specified. There will be no adjustment of the alpha level to account for multiple comparisons because the primary and secondary outcomes are specified, unless otherwise stated to be post hoc.

Study evaluation

A logic model for evaluation of this study is presented in **Figure 2**. The model summarises the activities involved in the development and implementation phases, as well as the projected outcomes. The model will be used to frame our approach to evaluating (1) whether the trial was developed and implemented as intended (fidelity and reach), (2) whether study outcomes were achieved or not, (3) the influence of situational factors on results obtained. Baseline surveys, results from the screening and QUM services, and pharmacists' interviews will be used to assess these activities.

Baseline evaluation

At the beginning of the study, participating pharmacies will be required to complete a baseline survey that includes questions on pharmacy characteristics, existing professional services (remunerated and non-remunerated), and strategies and capacity for implementing these services. The information collected will be used to inform the comparability of the two arms in the trial and health economic analysis. All data entered by participant pharmacists into the study software will be extracted to generate quantitative data to enable the trial process evaluation.

Impact evaluation

For peer review only - http://bmjopen.bmj.com/site/about/guidelines.xhtml

The impact of the screening and QUM services will be determined by testing the clinical hypothesis as detailed in the clinical protocol and outcomes sections.

Midtrial interviews

Three months after the implementation of the service in the pharmacy, a mix of pharmacists from high and low performance groups in both arms will be invited to participate in an interview to provide feedback to identify challenges, opportunities, barriers, and facilitators to service implementation. This will enable us to provide support to pharmacies and adapt where appropriate.

End-of-trial interviews

At the end of the study, pharmacists from participating pharmacies representing a mixture of both high recruitment and low recruitment, will be interviewed to gain feedback on the service implementation in the pharmacy, identifying barriers and facilitators to implementing the trial in the pharmacy, perceptions of sustainability and level of engagement by the local GPs in the referral process.

All study participants will receive a survey to determine their quality of life and wellbeing. Referred participants will also receive a survey to determine their level of satisfaction and willingness to pay for the provided service. A random sample of 20% non-referred participants will be surveyed to determine their overall experience and level of satisfaction with the service. A random sample of 10% of GPs who had patients referred to them during the trial will be invited to respond to a telephone/electronic questionnaire to determine their overall experience and level of satisfaction with the service.

All surveys will be created on Research Electronic Data Capture (REDCap), a secure web-based application designed to support data capture for research studies hosted at The University of Sydney [43]. Qualitative interviews will comprise open-ended questions that prompt discussion on a wide range of topics including overall experience with the study, consumer feedback, interaction with GPs and impact of the trial in their pharmacy.

Economic Evaluation

Health service utilisation will be measured through Medicare and Pharmaceutical Benefits Schemes (MBS and PBS) data linkage, to quantify the symptom-specific and total health service resource use and costs at 12 months, by allocated group. The Consolidated Health Economic Evaluation Reporting Standards (CHEERS) guidelines (**Appendix 3**) will be followed when reporting the health economic evaluation [44].

For peer review only - http://bmjopen.bmj.com/site/about/guidelines.xhtml

Costs

Cost-effectiveness of the intervention will be calculated from a health service provider perspective considering costs incurred by the pharmacists and the government (PBS/MBS) but will not include costs borne by screening participants (e.g., through co-payments charged by GPs). Healthcare resource use including GP visits, specialist visits, diagnostic tests, medicine, hospitalization, emergency admissions will be collected from MBS/PBS history, medication prescription and dispense records, and previous hospital discharge summaries from My Health Record. Unit costs will be collected based on MBS/PBS item numbers, Australian Refined Diagnosis Related Groups (AR-DRG) and Triage codes. Measured costs will include pharmacist time to facilitate data collection at each pharmacy.

Costs analysis

The cost per new CKD case diagnosis for each group will be estimated and compared. We will assess whether the average cost of the new CKD case diagnosis in the intervention group is lower or higher than the average cost of the new CKD case diagnosis in control group. We hypothesise that there is likely to be a difference in average costs per new CKD case diagnosis across the sites in the same cluster and across the clusters from the metropolitan and rural settings. This is due to the differences in sample size, local unit prices of equipment, staff costs and different established local practices.

Differences in overall mean costs between the two groups will be analysed using Generalised Linear Mixed Model. The distribution of residuals from the regression model will be examined and a decision will be made as to whether the costs should be logarithm transformed. Any extreme costs will be carefully examined to decide if they should be considered as extreme outliers and should be trimmed (removed) from the analysis. It is anticipated there will be some missing data. Missing costs data will be examined to ascertain whether it is missing completely at random (MCAR), missing at random (MAR), or missing not at random (MNAR). Simple and multiple imputation methods will be considered based on the pattern of missingness [45].

Effectiveness

Based on the EQ-5D-5L, utility weight will be calculated via Australian algorithm, which will be used to obtain quality adjusted life-years (QALY).

Within-trial cost-effectiveness analysis

Incremental cost effectiveness ratios (ICERs) that allow analysis of incremental costs of intervention group versus control group per one QALY gained will be calculated.

For peer review only - http://bmjopen.bmj.com/site/about/guidelines.xhtml

For peer review only

Modelled cost-effectiveness analysis

The modelled economic evaluation would investigate the benefits of early diagnosis of CKD cases and the associated prevention/delay of the CKD complications like use of kidney replacement therapies or mortality in the long term. The modelled economic evaluation will include the long-term costs of treatment, regular laboratory investigations, treatment of adverse events, hospital admissions and other relevant costs.

The long-term improved outcomes are hypothesised to translate into a cost reduction and offset the increased costs associated with adding pharmacy-led screening to no screening, for CKD case diagnosis. The modelled economic evaluation will also use published results of long-term observational studies to derive assumptions for the decision analytic modelling and to populate the model. A 5% discount rate will be applied to both costs and benefits [46]. The uncertainty about results of economic evaluation will be explored using a non-parametric bootstrapping method.

Patient and public involvement

The study will engage key stakeholders in designing, conducting, and reporting the research, as shown in Figure 2. Input from consumers and consumer organisations will be actively sought throughout the study implementation. This will be achieved by involving consumers and organisations, such as Kidney Health Australia and the Pharmaceutical Society of Australia, in both the project steering committee and the expert advisory group. Their roles will include contributing to the development of training materials for pharmacists, advising on effective communication and engagement strategies with pharmacists and GPs, and facilitating the implementation and dissemination of research progress and findings via newsletters.

Ethics and dissemination

The study will be conducted in compliance with the International Conference on Harmonisation Good Clinical Practice guidelines, the World Medical Declaration of Helsinki and all amendments, and the Protocol and Protocol Amendments.

The protocol, participant information sheets, consent forms and other relevant case report forms have all been approved by The University of Sydney Human Research Ethics Committee (2022/044). The trial protocol is also registered with the Australian New Zealand Clinical Trials Registry (ACTRN12622000329763) [47]. The trial registration is provided in **Appendix 4**. Study findings will be disseminated in plain language and peer-reviewed forums, including

For peer review only

For peer review only - http://bmjopen.bmj.com/site/about/guidelines.xhtml

at conferences, via publication in medical journals, and to participants, study staff, clinicians, and patient groups, via direct approaches and a variety of traditional and electronic media.

DISCUSSION

The proposed study presents a protocol for the first large-scale cluster randomised trial to evaluate the effectiveness of community pharmacy-led CKD screening and medication management in improving early disease detection and QUM. The sampling plan primarily targets kidney disease hotspot areas identified by Kidney Health Australia, but also accounts for access to services (i.e., metropolitan and rural locations). Within Australia, socio-economic status is lower in rural and remote areas relative to metropolitan areas [33]. The inclusion of MM2-MM5 locations is also relevant to First Nations communities – these areas overlap much of Inner and Outer Australia where 44% of First Nations people reside [33, 48]. The study is expected to raise awareness among both pharmacists and at-risk individuals about CKD and its risk factors, potentially leading to the early detection of previously unknown CKD cases. Further, by promoting the safe and effective use of medicines, the study findings will potentially lead to reduced adverse drug events and medication-related hospital admissions in people at risk of, or who live with, CKD. Collectively, the study will generate evidence to support the translation of new preventive and public health measures into practice and implement an innovative approach to improving the quality and cost-effectiveness of preventive healthcare intervention.

Effective and sustainable primary care interventions aimed at the early detection and management of CKD have the potential to not only improve health outcomes but also significantly reduce costs associated with extensive hospital services required in later stages, mainly due to the need for regular dialysis or a kidney transplant, which is estimated to cost the Australian economy $5.7 billion a year [49]. More importantly, recent modelling has shown that investing in early detection of kidney disease can save the Australian economy about $10.2 billion over the next 20 years [49]. Pharmacists are well placed to play a role in the early detection, education, and referral of individuals at elevated risk of CKD. By offering screening and QUM services, community pharmacists can contribute to health promotion and education. As integral members of primary healthcare, community pharmacists serve as an important channel for delivering these types of services. Further, their involvement in CKD care contributes towards improving the health status of Australians, including those from lower socioeconomic groups who are disproportionately affected by CKD; increasing public knowledge and awareness of CKD and its risk factors; expanding public health roles for

For peer review only - http://bmjopen.bmj.com/site/about/guidelines.xhtml

community pharmacists; and enhancing the roles of pharmacists as an integral part of the health workforce. This aligns with Australia's Primary Health Care 10 Year Plan, which promotes better utilisation of primary health care workforce, including allied health professionals and pharmacists [50].

There is widespread agreement that an effective program is needed to support the identification and better management of individuals with early stages of CKD. The subsequent adoption of such a program, if shown to be successful on a larger scale and in routine practice in Australia, has enormous potential to reduce the population levels of risk and hence reduce preventable healthcare expenditures due to the need for expensive treatments in advanced disease. The adoption of the program will also enhance the effectiveness of currently remunerated pharmacists' QUM services such as MedsCheck and Diabetes MedsCheck, which aim to improve patients' understanding of their medications and ultimately patient outcomes.

By targeting a representative sample of community pharmacies matched by location (rurality) and socioeconomic status [51], the study is anticipated to represent a wide range of demographics and regions in Australia. Some of the limitations anticipated during recruitment have been previously described and, while it may not be possible to avoid them fully, we will implement proper incentives to the participating pharmacies, pharmacists, and GPs. One major limitation of implementing such programmes is the lack of kidney function information availability, which impacts pharmacist's ability to identify and intervene in medication-related problems in people with CKD. Using POCT for determining kidney function markers in community pharmacies, the proposed study aims to fill this gap and promote pharmacists' contributions to CKD care. Finally, the high staff turnover rates and uneven distribution of the pharmacy workforce based on remoteness may affect the recruitment of pharmacists and patients in certain settings, particularly in remote and rural areas [52, 53].

This trial introduces an innovative approach by leveraging existing healthcare resources like My Health Record for a more streamlined referral and medication management purposes. This will improve the utilisation of available resources and maximises their potential. Further, the trial employs an integration-based software system that works with selected pharmacy dispensing systems. This integration is expected to provide a more efficient decision support system for medication review and referral, contributing to improved overall patient care. In addition to exploring community pharmacies' readiness to incorporate technological solutions

For peer review only - http://bmjopen.bmj.com/site/about/guidelines.xhtml

to address QUM issues, the study will shed light on potential barriers and enablers to scalability and sustainability of such systems.

In summary, this research presents a unique opportunity for community pharmacists to engage in health promotion and education of people who are at risk of or living with CKD. Given current existing chronic disease services in community pharmacies, our research will incorporate pharmacists in CKD screening and ensure quality medicines use in people who are at risk of or living with CKD. This is in line with the recommended clinical practice guidelines and National Strategic Plan that highlight the importance of early identification and intervention to slow the progression of CKD and occurrence of complications through a multidisciplinary primary care approach [2, 54].

FUNDING. This work was supported by the Medical Research Future Fund of the Australian Government primary healthcare initiative (MRFQI00008) and conducted collaboratively between The Universities of Sydney, Melbourne, Queensland, and Kidney Health Australia. The funding body has no role in the design of the study as well as collection, analysis and interpretation of data and manuscript write-up.

COMPETING INTERESTS. David Johnson has received consultancy fees, research grants, speaker's honoraria and travel sponsorships from Baxter Healthcare and Fresenius Medical Care, consultancy fees from Astra Zeneca, Bayer, and AWAK, speaker's honoraria from ONO and Boehringer Ingelheim & Lilly, and travel sponsorships from Ono and Amgen. He is a current recipient of an Australian National Health and Medical Research Council Leadership Investigator Grant. Kamal Sud has received consultancy fees and speaker's honoraria from Baxter Healthcare and speaker's honoraria from Roche. He is on the medical advisory board of Fresenius Medical Care for Australia and New Zealand.

CONTRIBUTORSHIP. WT & RC drafted the manuscript. WT, IK, KS, DJ, CV, RM, JM, AT, SV, LK, NG, RC were involved in the study concept and design. VV developed the geographical sampling frame and randomisation postcodes. JF developed the statistical analysis plan, while AT developed the health economics evaluation plan. WT, IK, KS, DJ, CV, VV, RM, JF, JM, AT, BR, SV, LK, NG, MF, VT, NC, RC critically reviewed the trial protocol. All authors have read, revised, and approved the final manuscript.

For peer review only

REFERENCES

1. Hill, N.R., et al., *Global prevalence of chronic kidney disease–a systematic review and meta-analysis.* PloS one, 2016. **11**(7): p. e0158765.
2. *Kidney Health Australia 2019. National Strategic Action Plan for Kidney Disease.* 20/03/2023]; Available from: https://kidney.org.au/get-involved/advocacy/national-strategic-action-plan-for-kidney-disease.
3. Cockwell, P. and L.-A. Fisher, *The global burden of chronic kidney disease.* Lancet, 2020. **395**(10225): p. 662-664.
4. Fink, J., et al., *Finding a common language for patient safety in CKD.* Clin J Am Soc Nephrol, 2012. **7**(4): p. 689-695.
5. Hu KT, M.A., Stevenson FT, *Calculation of the estimated creatinine clearance in avoiding drug dosing errors in the older patient.* Am J Med Sci, 2001. **322**(3): p. 133-136.
6. Khanal A, P.G., Castelino RL, Jose MD, *Potentially inappropriate prescribing of renally cleared drugs in elderly patients in community and aged care settings*
Drugs Aging, 2015. **32**(5): p. 391-400.
7. Long CL, R.M., Price DW, Magid DJ, *Compliance with dosing guidelines in patients with chronic kidney disease.* Ann Pharmacother, 2004. **38**(5): p. 853-858.
8. Papaioannou A, C.J., Campbell G, Bedard M, *Assessment of adherence to renal dosing guidelines in long-term care facilities.* J Am Geriatr Soc, 2000. **48**(11): p. 1470-1473.
9. Yap C, D.D., Thompson J, Baker D, *Medication dosing errors for patients with renal insufficiency in ambulatory care.* Jt Comm J Qual Patient Saf, 2005. **31**(9): p. 514-521.
10. Castelino, R., et al., *Quality Use Of Medicines In Patients With Chronic Kidney Disease.* BMC Nephrol, 2020. **21**(1): p. 216.
11. Doody HK, P.G., Watson D, Castelino RL, *Retrospective evaluation of potentially inappropriate prescribing in hospitalized patients with renal impairment.* Curr Med Res Opin, 2015. **31**(3): p. 525-535.
12. Tesfaye, W.H., et al., *Inappropriate prescribing in chronic kidney disease: A systematic review of prevalence, associated clinical outcomes and impact of interventions.* Int J Clin Pract, 2017. **71**(7): p. e12960.
13. Kitsos, A., Peterson, G., Jose, M., Khanam, M., Castelino, R., Radford, J, *Variation in Documenting Diagnosable Chronic Kidney Disease in General Medical Practice:*

For peer review only - http://bmjopen.bmj.com/site/about/guidelines.xhtml

Implications for Quality Improvement and Research. J Prim Care Community Health, 2019.

14. *Kidney Health Australia (KHA). State of the Nation 2016. Kidney Health Week. Chronic Kidney Disease Hot Spots*. 2016 2/2/2021]; Available from: https://kidney.org.au/uploads/resources/state-of-the-nation-kidney-health-week-2016-chronic-kidney-disease-hot-spots.pdf
15. Fox CH, B.A., Zayas LE, McClellan W, Murray B. , *Primary care physicians' knowledge and practice patterns in the treatment of chronic kidney disease: an Upstate New York Practice-based Research Network (UNYNET) study.* J Am Board Fam Med, 2006. **19**(1): p. 54-61.
16. Johnson, D., *Evidence-based guide to slowing the progression of early renal insufficiency.* Intern Med J, 2004. **34**(1-2): p. 50-57.
17. Nagykaldi, Z., J. Mold, and C. Aspy, *Practice facilitators: a review of the literature.* Fam Med, 2005. **37**(8): p. 581-588.
18. Duckett, S. and D. Romanes, *Submission to Review of Pharmacy Remuneration and Regulation*, in *Grattan Institute*. 2016.
19. *NAB Pharmacy Survey*. 2021 12/05/2023]; Available from: https://www.guild.org.au/__data/assets/pdf_file/0014/112055/NAB-Pharmacy-Report-2021.pdf.
20. Hendrie, D., *Australians are visiting their GP more and more*, in *newsGP*. 2018.
21. Mc Namara, K., H. Alzubaidi, and J.K. Jackson, *Cardiovascular disease as a leading cause of death: how are pharmacists getting involved?* Integr Pharm Res Pract, 2019. **8**: p. 1-11.
22. San-Juan-Rodriguez, A., et al., *Impact of community pharmacist-provided preventive services on clinical, utilization, and economic outcomes: An umbrella review.* Prev Med, 2018. **115**: p. 145-155.
23. Krass, I., et al., *Pharmacy Diabetes Screening Trial: protocol for a pragmatic clusterrandomised controlled trial to compare three screening methods for undiagnosed type 2 diabetes in Australian community pharmacy.* BMJ Open, 2017.
24. Jackson SL, P.G., House M, Bartlett T, *Point‐of‐care monitoring of anticoagulant therapy by rural community pharmacists: Description of successful outcomes.* Aust J Rural Health, 2004. **12**(5): p. 197-200.

For peer review only - http://bmjopen.bmj.com/site/about/guidelines.xhtml

25. Krass, I., et al., *Pharmacy diabetes screening trial (PDST): Outcomes of a national clustered RCT comparing three screening methods for undiagnosed type 2 diabetes (T2DM) in community pharmacy.* Diabetes Res Clin Pract, 2023. **197**: p. 110566.

26. Geerts, A.F., et al., *Feasibility of point-of-care creatinine testing in community pharmacy to monitor drug therapy in ambulatory elderly patients.* J Clin Pharm Ther, 2013. **38**(5): p. 416-22.

27. Donovan, J., et al., *Community pharmacist identification of chronic kidney disease using point-of-care technology: A pilot study.* Can Pharm J, 2020. **153**(2): p. 84-87.

28. Escribá-Martí, G., et al., *Pharmaceutical care program for patients with chronic kidney disease in the community pharmacy: Detection of nephrotoxic drugs and dose adjustment. Viability study.* PLoS One, 2022. **17**(12): p. e0278648.

29. Gheewala, P.A., et al., *Evaluation of a chronic kidney disease risk assessment service in community pharmacies.* Nephrology, 2019. **24**(3): p. 301-307.

30. Gheewala, P., et al., *Australian Community Pharmacists' Experience of Implementing a Chronic Kidney Disease Risk Assessment Service.* . Prev Chronic Dis, 2018. **15**: p. e81.

31. Chan, A.-W., et al., *SPIRIT 2013 Statement: Defining Standard Protocol Items for Clinical Trials.* Ann Intern Med, 2013. **158**(3): p. 200-207.

32. *Socio-Economic Indexes for Areas*. 2016 3/02/2021]; Available from: https://www.abs.gov.au/ausstats/abs@.nsf/mf/2033.0.55.001.

33. Versace, V.L., et al., *National analysis of the Modified Monash Model, population distribution and a socio-economic index to inform rural health workforce planning.* Aust J Rural Health, 2021. **29**(5): p. 801-810.

34. Beks, H., et al., *Approaches used to describe, measure, and analyze place of practice in dentistry, medical, nursing, and allied health rural graduate workforce research in Australia: a systematic scoping review.* Int J Environ Res Public Health, 2022. **19**(3): p. 1438.

35. *GRT Sample Size Calculator - Research Methods Resources | National Institutes of Health (NIH)*. 2021 3/2/2021]; Available from: https://researchmethodsresources.nih.gov/rmr-calculator/build/#/step1

36. *Nova Biomedical. Novo StatSensor. Point of Care Creatinine and eGFR Analyzer.* 2021; Available from: http://www.novabio.us/statstrip-creatinine/

For peer review only - http://bmjopen.bmj.com/site/about/guidelines.xhtml

37. Blairon, L., et al., *Improving NSAIDs Prescription in Emergency Services Unit by a Point-of-Care-Based Renal Function Evaluation.* J Emerg Med, 2020. **58**(3): p. 481-486.

38. *QKidney® - Risk Calculator*. 2018 12/05/2023]; Available from: http://www.qkidney.org/index.php

39. Herdman M, G.C., Lloyd A, Janssen M, Kind P, Parkin D, Bonsel G, Badia X, *Development and preliminary testing of the new five-level version of EQ-5D (EQ-5D-5L).* Qual Life Res, 2011. **20**: p. 1727-36.

40. Norman R, C.P., Viney R, *A pilot discrete choice experiment to explore preferences for EQ-5D-5L health states.* Appl Health Econ Health Policy, 2013. **11**(287-98).

41. Walters SJ, B.J., *Comparison of the minimally important difference for two health state utility measures: EQ-5D and SF-6D.* Qual Life Res, 2005. **14**(6): p. 1523-32.

42. Moher, D., et al., *CONSORT 2010 explanation and elaboration: updated guidelines for reporting parallel group randomised trials.* J Clin Epidemiol, 2010. **63**(8): p. e1-37.

43. Harris PA, T.R., Thielke R, et al, *Research electronic data capture (REDCap)–a metadata-driven methodology and workflow process for providing translational research informatics support.* J Biomed Inform, 2009. **42**: p. 377-81.

44. Husereau, D., et al., *Consolidated Health Economic Evaluation Reporting Standards (CHEERS) statement.* Bmj, 2013. **346**: p. f1049.

45. Glick, H.A., et al., *Economic evaluation in clinical trials*. 2014: OUP Oxford.

46. Australia. Department of Health, H. and S. Community, *Guidelines for the pharmaceutical industry on preparation of submissions to the Pharmaceutical Benefits Advisory Committee, including submissions involving economic analyses* 1992, Canberra: Commonwealth Department of Health, Housing and Community Services.

47. *Pharmacy-Based Screening and Quality Use of Medicines in Kidney Disease*. 2022 05/04/2022]; Available from: https://www.anzctr.org.au/Trial/Registration/TrialReview.aspx?id=383269&isClinicalTrial=False.

48. Beks, H., et al., *Geographical analysis of evaluated chronic disease programs for Aboriginal and Torres Strait Islander people in the Australian primary health care setting: a systematic scoping review.* BMC Public Health, 2019. **19**(1): p. 1115.

For peer review only

49. *Changing the chronic kidney disease landscape: The economic benefits of early detection and treatment*. 2023 03/07/2023]; Available from: https://www2.deloitte.com/content/dam/Deloitte/au/Documents/Economics/deloitte-au-economics-kidney-health-australia-report-80323.pdf.

50. *Future focused primary health care: Australia's Primary Health Care 10 Year Plan 2022-2032* 2022 01/08/2023]; Available from: https://www.health.gov.au/sites/default/files/documents/2022/03/australia-s-primary-health-care-10-year-plan-2022-2032-future-focused-primary-health-care-australia-s-primary-health-care-10-year-plan-2022-2032.pdf.

51. Statistics., A.B.o. *Socio-Economic Indexes for Areas*. 2016 3/02/2021]; Available from: https://www.abs.gov.au/ausstats/abs@.nsf/mf/2033.0.55.001.

52. Thin, S.M., et al., *A systematic review on pharmacists' turnover and turnover intention.* Res Social Adm Pharm, 2022. **18**(11): p. 3884-3894.

53. Obamiro, K., T. Barnett, and I. Inyang, *The Australian pharmacist workforce: distribution and predictors of practising outside of metropolitan and regional areas in 2019.* Int J Pharm Pract, 2022. **30**(4): p. 354-359.

54. James, M.T., B.R. Hemmelgarn, and M. Tonelli, *Early recognition and prevention of chronic kidney disease.* Lancet, 2010. **375**(9722): p. 1296-1309.

For peer review only

For peer review only - http://bmjopen.bmj.com/site/about/guidelines.xhtml

FIGURE LEGENDS

Figure 1. Procedures of recruitment of participants.

Figure 2. Pharmacy based screening and QUM study logic model.

For peer review only

For peer review only - http://bmjopen.bmj.com/site/about/guidelines.xhtml

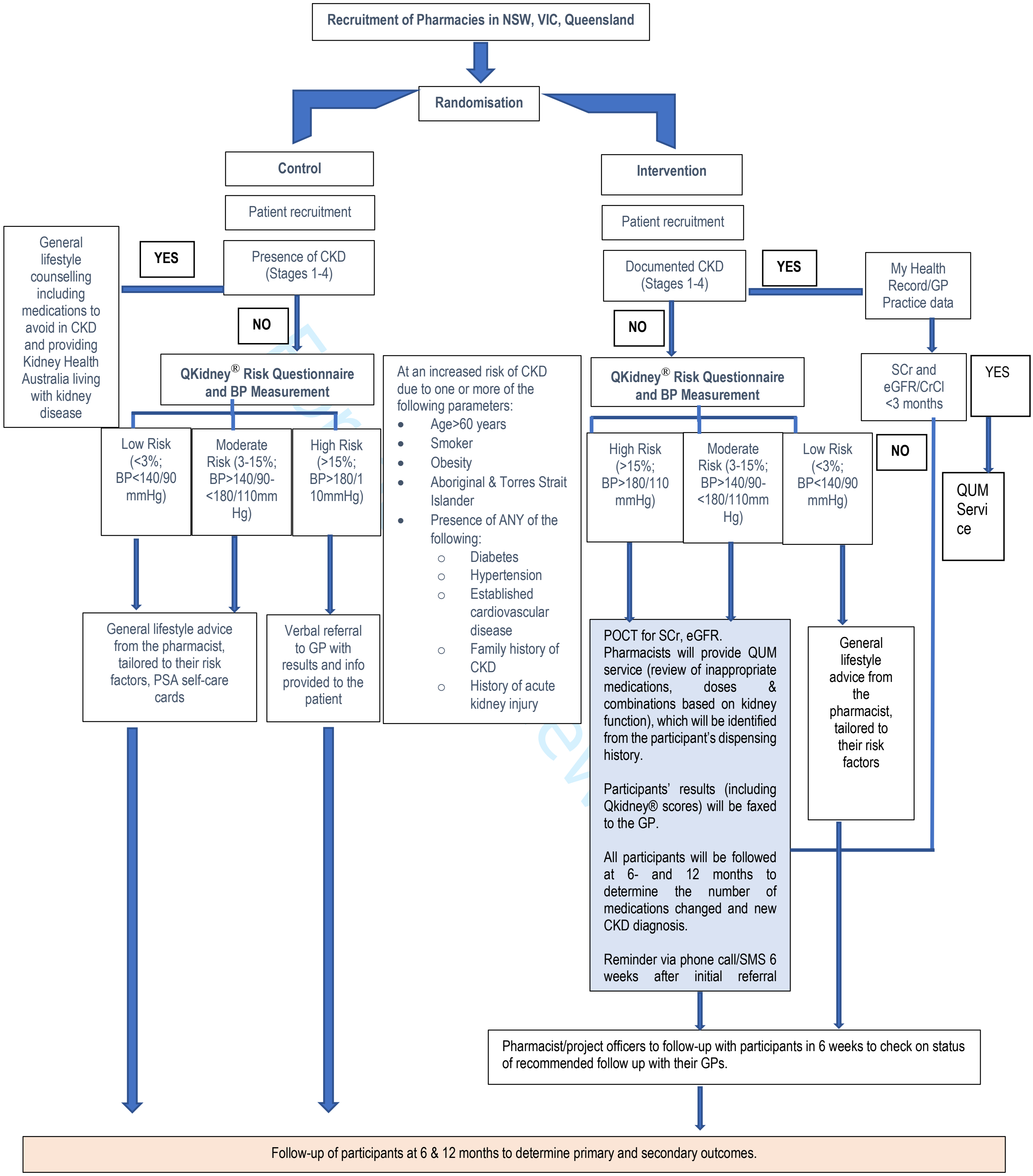

Recruitment of Pharmacies in NSW, VIC, Queensland
Randomisation
Control
Patient recruitment
Presence of CKD (Stages 1-4)
YES
General lifestyle counselling including medications to avoid in CKD and providing Kidney Health Australia living with kidney disease
NO
QKidney® Risk Questionnaire and BP Measurement
Low Risk (<3%; BP<140/90 mmHg)
Moderate Risk (3-15%; BP>140/90-<180/110mm Hg)
High Risk (>15%; BP>180/110mmHg)
General lifestyle advice from the pharmacist, tailored to their risk factors, PSA self-care cards
Verbal referral to GP with results and info provided to the patient
At an increased risk of CKD due to one or more of the following parameters:
Age>60 years
Smoker
Obesity
Aboriginal & Torres Strait Islander
Presence of ANY of the following:
Diabetes
Hypertension
Established cardiovascular disease
Family history of CKD
History of acute kidney injury
Intervention
Patient recruitment
Documented CKD (Stages 1-4)
YES
My Health Record/GP Practice data
SCr and eGFR/CrCl <3 months
YES
QUM Service
NO
NO
QKidney® Risk Questionnaire and BP Measurement
High Risk (>15%; BP>180/110 mmHg)
Moderate Risk (3-15%; BP>140/90-<180/110mm Hg)
Low Risk (<3%; BP<140/90 mmHg)
POCT for SCr, eGFR. Pharmacists will provide QUM service (review of inappropriate medications, doses & combinations based on kidney function), which will be identified from the participant's dispensing history.
Participants' results (including Qkidney® scores) will be faxed to the GP.
All participants will be followed at 6- and 12 months to determine the number of medications changed and new CKD diagnosis.
Reminder via phone call/SMS 6 weeks after initial referral
General lifestyle advice from the pharmacist, tailored to their risk factors
Pharmacist/project officers to follow-up with participants in 6 weeks to check on status of recommended follow up with their GPs.
Follow-up of participants at 6 & 12 months to determine primary and secondary outcomes.

INPUTS	ACTIVITIES	OUTPUTS	SHORT-TERM OUTCOMES	ANTICIPATED LONG-TERM OUTCOMES
**User and Stakeholders**<br>Consumers (patients)<br>Community pharmacists<br>Community pharmacy staff<br>GPs and practice staff<br><br>**Technology Components**<br>Electronic templates for screening and QUM services, including referral templates, Qkidney® risk and results.<br><br>**Training**<br>Online CPD training program developed for participating pharmacists.<br><br>**Other resources**<br>Material resources (e.g., promotional material (flyers, posters for display etc), Stat sensor POCT for SCr and eGFR/CrCL.	**Preliminary**<br>Project steering committee and Expert panel convened to provide advice on trial implementation; and how to support engagement with GPs, Primary Health Networks, recruit, retain participants, and community pharmacies.<br><br>Recruit pharmacies.<br><br>Implementation of the cRCT.<br><br>**Pharmacists' intervention**<br>Initial training.<br>Implementation planning.<br>Recruitment of eligible participants.<br>Referral of participants with undiagnosed CKD and/or inappropriate medications as per kidney function to the GP according to the clinical protocol.<br><br>Follow up of patients at 6 and 12 months.<br>Regular report of trial progress.<br><br>**Maintenance**<br>Maintain relationship with pharmacies, pharmacists, and other stakeholders.	Improved rates of detection of people with undiagnosed CKD<br><br>Improved use of medicines in patients with kidney disease<br><br>Improved awareness of lifestyle measures to reduce CKD risk.<br><br>Improved collaboration between pharmacists and GPs	Will a pharmacy-led screening and QUM service improve identification of undiagnosed CKD and improve the use of medications?<br><br>**Primary Outcomes**<br>Proportion of newly diagnosed CKD patients<br><br>Proportion of medication changes<br><br>**Secondary Outcomes**<br>Proportion of people with inappropriate medications<br><br>Proportion of people with chronic kidney disease<br><br>Proportion of people screened.<br><br>QKidney® risk profiles<br><br>% Of people referred to their GP<br><br>% Who take up referral with their GP.<br><br>Hospitalisations due to medication-related events.<br><br>Quality of life and other humanistic outcomes such as well-being and consumer satisfaction.<br><br>Cost effectiveness.	Reduced incidence of kidney failure.<br><br>Reduced medication-related events, hospital admissions and mortality related to CKD.<br><br>Guide health policy to efficient use of primary health care resources.

For peer review only

SPIRIT

STANDARD PROTOCOL ITEMS: RECOMMENDATIONS FOR INTERVENTIONAL TRIALS

Appendix 1: SPIRIT 2013 Checklist: Recommended items to address in a clinical trial protocol and related documents*

Section/item	Item No	Description	Addressed on page number
**Administrative information**			
Title	1	Descriptive title identifying the study design, population, interventions, and, if applicable, trial acronym	1
Trial registration	2a	Trial identifier and registry name. If not yet registered, name of intended registry	18
Protocol version	3	Date and version identifier	v2.0_14-02-22
Funding	4	Sources and types of financial, material, and other support	21
Roles and responsibilities	5a	Names, affiliations, and roles of protocol contributors	21
	5b	Name and contact information for the trial sponsor	21
	5c	Role of study sponsor and funders, if any, in study design; collection, management, analysis, and interpretation of data; writing of the report; and the decision to submit the report for publication, including whether they will have ultimate authority over any of these activities	21
	5d	Composition, roles, and responsibilities of the coordinating centre, steering committee, endpoint adjudication committee, data management team, and other individuals or groups overseeing the trial, if applicable (see Item 21a for data monitoring committee)	11 (governance details included in master protocol & available on request)

For peer review only

**Introduction**			
Background and rationale	6a	Description of research question and justification for undertaking the trial, including summary of relevant studies (published and unpublished) examining benefits and harms for each intervention	3-4
	6b	Explanation for choice of comparators	4-5
Objectives	7	Specific objectives or hypotheses	5
Trial design	8	Description of trial design including type of trial (eg, parallel group, crossover, factorial, single group), allocation ratio, and framework (eg, superiority, equivalence, noninferiority, exploratory)	5
**Methods: Participants, interventions, and outcomes**			
Study setting	9	Description of study settings (eg, community clinic, academic hospital) and list of countries where data will be collected. Reference to where list of study sites can be obtained	5-6
Eligibility criteria	10	Inclusion and exclusion criteria for participants. If applicable, eligibility criteria for study centres and individuals who will perform the interventions (eg, surgeons, psychotherapists)	7-9
Interventions	11a	Interventions for each group with sufficient detail to allow replication, including how and when they will be administered	9-11
	11b	Criteria for discontinuing or modifying allocated interventions for a given trial participant (eg, drug dose change in response to harms, participant request, or improving/worsening disease)	NA
	11c	Strategies to improve adherence to intervention protocols, and any procedures for monitoring adherence (eg, drug tablet return, laboratory tests)	11
	11d	Relevant concomitant care and interventions that are permitted or prohibited during the trial	NA
Outcomes	12	Primary, secondary, and other outcomes, including the specific measurement variable (eg, systolic blood pressure), analysis metric (eg, change from baseline, final value, time to event), method of aggregation (eg, median, proportion), and time point for each outcome. Explanation of the clinical relevance of chosen efficacy and harm outcomes is strongly recommended	12-13
Participant timeline	13	Time schedule of enrolment, interventions (including any run-ins and washouts), assessments, and visits for participants. A schematic diagram is highly recommended (see Figure 1)	12

For peer review only - http://bmjopen.bmj.com/site/about/guidelines.xhtml

Sample size	14	Estimated number of participants needed to achieve study objectives and how it was determined, including clinical and statistical assumptions supporting any sample size calculations	7
Recruitment	15	Strategies for achieving adequate participant enrolment to reach target sample size	4

Methods: Assignment of interventions (for controlled trials)

Allocation:			
Sequence generation	16a	Method of generating the allocation sequence (eg, computer-generated random numbers), and list of any factors for stratification. To reduce predictability of a random sequence, details of any planned restriction (eg, blocking) should be provided in a separate document that is unavailable to those who enrol participants or assign interventions	6
Allocation concealment mechanism	16b	Mechanism of implementing the allocation sequence (eg, central telephone; sequentially numbered, opaque, sealed envelopes), describing any steps to conceal the sequence until interventions are assigned	NA
Implementation	16c	Who will generate the allocation sequence, who will enrol participants, and who will assign participants to interventions	NA
Blinding (masking)	17a	Who will be blinded after assignment to interventions (eg, trial participants, care providers, outcome assessors, data analysts), and how	NA
	17b	If blinded, circumstances under which unblinding is permissible, and procedure for revealing a participant's allocated intervention during the trial	NA

Methods: Data collection, management, and analysis

Data collection methods	18a	Plans for assessment and collection of outcome, baseline, and other trial data, including any related processes to promote data quality (eg, duplicate measurements, training of assessors) and a description of study instruments (eg, questionnaires, laboratory tests) along with their reliability and validity, if known. Reference to where data collection forms can be found, if not in the protocol	11
	18b	Plans to promote participant retention and complete follow-up, including list of any outcome data to be collected for participants who discontinue or deviate from intervention protocols	11, 16-18

Data management	19	Plans for data entry, coding, security, and storage, including any related processes to promote data quality (eg, double data entry; range checks for data values). Reference to where details of data management procedures can be found, if not in the protocol	NA (included in the master protocol & available on request)
Statistical methods	20a	Statistical methods for analysing primary and secondary outcomes. Reference to where other details of the statistical analysis plan can be found, if not in the protocol	14-18
	20b	Methods for any additional analyses (eg, subgroup and adjusted analyses)	14-18
	20c	Definition of analysis population relating to protocol non-adherence (eg, as randomised analysis), and any statistical methods to handle missing data (eg, multiple imputation)	14-15
**Methods: Monitoring**			
Data monitoring	21a	Composition of data monitoring committee (DMC); summary of its role and reporting structure; statement of whether it is independent from the sponsor and competing interests; and reference to where further details about its charter can be found, if not in the protocol. Alternatively, an explanation of why a DMC is not needed	NA (included in the master protocol)
	21b	Description of any interim analyses and stopping guidelines, including who will have access to these interim results and make the final decision to terminate the trial	NA (included in the master protocol)
Harms	22	Plans for collecting, assessing, reporting, and managing solicited and spontaneously reported adverse events and other unintended effects of trial interventions or trial conduct	NA (included in the master protocol)
Auditing	23	Frequency and procedures for auditing trial conduct, if any, and whether the process will be independent from investigators and the sponsor	11
**Ethics and dissemination**			
Research ethics approval	24	Plans for seeking research ethics committee/institutional review board (REC/IRB) approval	18
Protocol amendments	25	Plans for communicating important protocol modifications (eg, changes to eligibility criteria, outcomes, analyses) to relevant parties (eg, investigators, REC/IRBs, trial participants, trial registries, journals, regulators)	18-19

Consent or assent	26a	Who will obtain informed consent or assent from potential trial participants or authorised surrogates, and how (see Item 32)	9
	26b	Additional consent provisions for collection and use of participant data and biological specimens in ancillary studies, if applicable	NA
Confidentiality	27	How personal information about potential and enrolled participants will be collected, shared, and maintained in order to protect confidentiality before, during, and after the trial	NA (included in the ethics, master protocol)
Declaration of interests	28	Financial and other competing interests for principal investigators for the overall trial and each study site	21
Access to data	29	Statement of who will have access to the final trial dataset, and disclosure of contractual agreements that limit such access for investigators	NA (included in the ethics, master protocol)
Ancillary and post-trial care	30	Provisions, if any, for ancillary and post-trial care, and for compensation to those who suffer harm from trial participation	NA
Dissemination policy	31a	Plans for investigators and sponsor to communicate trial results to participants, healthcare professionals, the public, and other relevant groups (eg, via publication, reporting in results databases, or other data sharing arrangements), including any publication restrictions	18-19
	31b	Authorship eligibility guidelines and any intended use of professional writers	NA
	31c	Plans, if any, for granting public access to the full protocol, participant-level dataset, and statistical code	22
**Appendices**			
Informed consent materials	32	Model consent form and other related documentation given to participants and authorised surrogates	Available on request.
Biological specimens	33	Plans for collection, laboratory evaluation, and storage of biological specimens for genetic or molecular analysis in the current trial and for future use in ancillary studies, if applicable	NA

For peer review only - http://bmjopen.bmj.com/site/about/guidelines.xhtml

*It is strongly recommended that this checklist be read in conjunction with the SPIRIT 2013 Explanation & Elaboration for important clarification on the items. Amendments to the protocol should be tracked and dated. The SPIRIT checklist is copyrighted by the SPIRIT Group under the Creative Commons "Attribution-NonCommercial-NoDerivs 3.0 Unported" license.

For peer review only

Australian Government

Department of Health
Therapeutic Goods Administration

Australian Register of Therapeutic Goods Certificate

Issued to

Australasian Medical & Scientific Ltd

for approval to supply

Instrument/analyser IVDs

**ARTG Identifier**	197482
**ARTG Start Date**	15/05/2012
**Product Category**	Medical Device Included - IVD Class 1
**GMDN**	CT943
**GMDN Term**	Instrument/analyser IVDs
**Intended Purpose**	Instruments used in the quantitative determination of blood parameters in a whole blood specimen including haemoglobin and haematocrit, glucose (including glucose analysis with critically ill patients e.g. ED, ICU, NICU), ketones, creatinine and lactate (including foetal scalp lactate analysis)

Manufacturer Details	Address	Certificate number(s)
Nova Biomedical Corporation	200 Prospect Street<br>WALTHAM , MASSACH USETTS , 02454<br>United States Of America	

ARTG Standard Conditions

The above Medical Device Included - IVD Class 1 has been entered on the Register subject to the following conditions:

- - The inclusion of the kind of device in the ARTG is subject to compliance with all conditions placed or imposed on the ARTG entry. Refer Part 4-5, Division 2 (Conditions) of the Therapeutic Goods Act 1989 and Part 5, Division 5.2 (Conditions) of the Therapeutic Goods (Medical Devices) Regulations 2002 for relevant information.
  - Breaching conditions of the inclusion related to the device of the kind may lead to suspension or cancellation of the ARTG entry; may be a criminal offence; and civil penalties may apply.

Products Covered by This Entry

1. Instrument/analyser IVDs

This entry: does not contain System(s)/Procedure Pack(s)

IVD Information

Name	Category Description
StatStrip Lactate Hospital Meter	Point of care testing
Stat Strip Xpress 2 Meter	Point of care testing
StatStrip Xpress-i Glucose and Ketone Meter	Point of care testing
StatStrip Xpress-i Glucose Meter	Point of care testing

Name	Category Description
StatStrip LAC/Hb/Hct Meter	Point of care testing
Nova Statsensor Creatinine Xpress Meter	Point of care testing
StatStrip Glucose Hospital Meter	Point of care testing
StatStrip Hb/Hct Meter	Point of care testing
StatStrip Xpress-i Lactate Meter	Point of care testing
Stat Profile Prime CCS Analyzer	Point of care testing
Nova Statsensor Creatinine Meter	Point of care testing
Stat Profile Prime ABG Analyzer	Point of care testing
StatStrip Glucose and Ketone Hospital Meter	Point of care testing
StatStrip Xpress2 LAC/Hb/Hct Meter	Point of care testing
Stat Profile Prime Plus Analyser	Point of care testing

Product Specific Conditions

No specific conditions have been recorded against this entry.

Therapeutic Goods Administration
PO Box 100, Woden ACT 2606 Australia
Phone: 1800 020 653
Email: info@tga.gov.au

ARTG Identifier: 197482
ARTG Start Date: 15/05/2012

Australian Government

Department of Health and Aged Care
Therapeutic Goods Administration

Australian Register of Therapeutic Goods Certificate

Issued to

Australasian Medical & Scientific Ltd

for approval to supply

Instrument/analyser IVDs

**ARTG Identifier**	427409
**ARTG Start Date**	6/11/2023
**Product Category**	Medical Device Included - IVD Class 1
**GMDN**	CT943
**GMDN Term**	Instrument/analyser IVDs
**Intended Purpose**	Instruments used for in vitro diagnostic use for the quantitative measurement of creatinine and for calculating Estimated Glomerular Filtration Rate (eGFR).

Manufacturer Details	Address	Certificate number(s)
Nova Biomedical Corporation	200 Prospect Street<br>WALTHAM , MASSACH<br>USETTS , 02454<br>United States Of America	

ARTG Standard Conditions

The above Medical Device Included - IVD Class 1 has been entered on the Register subject to the following conditions:

· - The inclusion of the kind of device in the ARTG is subject to compliance with all conditions placed or imposed on the ARTG entry. Refer Part 4-5, Division 2 (Conditions) of the Therapeutic Goods Act 1989 and Part 5, Division 5.2 (Conditions) of the Therapeutic Goods (Medical Devices) Regulations 2002 for relevant information.
- Breaching conditions of the inclusion related to the device of the kind may lead to suspension or cancellation of the ARTG entry; may be a criminal offence; and civil penalties may apply.

Products Covered by This Entry

1. Instrument/analyser IVDs

This entry: does not contain System(s)/Procedure Pack(s)

IVD Information

Name	Category Description
Nova Max Pro Creatinine and eGFR Meter	Point of care testing

Product Specific Conditions

No specific conditions have been recorded against this entry.

Therapeutic Goods Administration
PO Box 100, Woden ACT 2606 Australia
Phone: 1800 020 653
Email: info@tga.gov.au

ARTG Identifier: 427409
ARTG Start Date: 6/11/2023

Appendix 3. CHEERS checklist

CHEERS Checklist
Items to include when reporting economic evaluations of health interventions

The **ISPOR CHEERS Task Force Report**, *Consolidated Health Economic Evaluation Reporting Standards (CHEERS)—Explanation and Elaboration: A Report of the ISPOR Health Economic Evaluations Publication Guidelines Good Reporting Practices Task Force*, provides examples and further discussion of the 24-item CHEERS Checklist and the CHEERS Statement. It may be accessed via the *Value in Health* or via the ISPOR Health Economic Evaluation Publication Guidelines – CHEERS: Good Reporting Practices webpage: http://www.ispor.org/TaskForces/EconomicPubGuidelines.asp

Section/item	Item No	Recommendation	Reported on page No/ line No
**Title and abstract**			
Title	1	Identify the study as an economic evaluation or use more specific terms such as "cost-effectiveness analysis", and describe the interventions compared.	
Abstract	2	Provide a structured summary of objectives, perspective, setting, methods (including study design and inputs), results (including base case and uncertainty analyses), and conclusions.	
**Introduction**			
Background and objectives	3	Provide an explicit statement of the broader context for the study. Present the study question and its relevance for health policy or practice decisions.	
**Methods**			
Target population and subgroups	4	Describe characteristics of the base case population and subgroups analysed, including why they were chosen.	
Setting and location	5	State relevant aspects of the system(s) in which the decision(s) need(s) to be made.	
Study perspective	6	Describe the perspective of the study and relate this to the costs being evaluated.	
Comparators	7	Describe the interventions or strategies being compared and state why they were chosen.	
Time horizon	8	State the time horizon(s) over which costs and consequences are being evaluated and say why appropriate.	
Discount rate	9	Report the choice of discount rate(s) used for costs and outcomes and say why appropriate.	
Choice of health outcomes	10	Describe what outcomes were used as the measure(s) of benefit in the evaluation and their relevance for the type of analysis performed.	
Measurement of effectiveness	11a	*Single study-based estimates:* Describe fully the design features of the single effectiveness study and why the single study was a sufficient source of clinical effectiveness data.	

Section/item	Item No	Recommendation	Reported on page No/ line No
	11b	*Synthesis-based estimates:* Describe fully the methods used for identification of included studies and synthesis of clinical effectiveness data.	
Measurement and valuation of preference based outcomes	12	If applicable, describe the population and methods used to elicit preferences for outcomes.	
Estimating resources and costs	13a	*Single study-based economic evaluation:* Describe approaches used to estimate resource use associated with the alternative interventions. Describe primary or secondary research methods for valuing each resource item in terms of its unit cost. Describe any adjustments made to approximate to opportunity costs.	
	13b	*Model-based economic evaluation:* Describe approaches and data sources used to estimate resource use associated with model health states. Describe primary or secondary research methods for valuing each resource item in terms of its unit cost. Describe any adjustments made to approximate to opportunity costs.	
Currency, price date, and conversion	14	Report the dates of the estimated resource quantities and unit costs. Describe methods for adjusting estimated unit costs to the year of reported costs if necessary. Describe methods for converting costs into a common currency base and the exchange rate.	
Choice of model	15	Describe and give reasons for the specific type of decision-analytical model used. Providing a figure to show model structure is strongly recommended.	
Assumptions	16	Describe all structural or other assumptions underpinning the decision-analytical model.	
Analytical methods	17	Describe all analytical methods supporting the evaluation. This could include methods for dealing with skewed, missing, or censored data; extrapolation methods; methods for pooling data; approaches to validate or make adjustments (such as half cycle corrections) to a model; and methods for handling population heterogeneity and uncertainty.	
**Results**			
Study parameters	18	Report the values, ranges, references, and, if used, probability distributions for all parameters. Report reasons or sources for distributions used to represent uncertainty where appropriate. Providing a table to show the input values is strongly recommended.	
Incremental costs and outcomes	19	For each intervention, report mean values for the main categories of estimated costs and outcomes of interest, as well as mean differences between the comparator groups. If applicable, report incremental cost-effectiveness ratios.	
Characterising uncertainty	20a	*Single study-based economic evaluation:* Describe the effects of sampling uncertainty for the estimated incremental cost and incremental effectiveness parameters, together with the impact	

Appendix 7. Trial registration

Status: Registered	Prospectively registered	Up to date

The impact of pharmacy-based screening on identification of unknown kidney disease and a quality use of medicines intervention on inappropriate medication use in people at risk for or living with chronic kidney disease

Trial Id: ACTRN12622000329763	Request Id: 383269	Update Trial	View Trial

Date Registered: 22/02/2022	Start date: 01/09/2022	Last approved: 22/02/2022

View data sharing statement
